# Postnatal prebiotic supplementation in rats affects adult anxious behaviour, hippocampus, electrophysiology, metabolomics, and gut microbiota

**DOI:** 10.1016/j.isci.2021.103113

**Published:** 2021-09-10

**Authors:** Sonia O. Spitzer, Andrzej Tkacz, Helene M. Savignac, Matthew Cooper, Natasa Giallourou, Edward O. Mann, David M. Bannerman, Jonathan R. Swann, Daniel C. Anthony, Philip S. Poole, Philip W.J. Burnet

**Affiliations:** 1 Department of Psychiatry, University of Oxford, Warneford Lane, Oxford, OX3 7JX, UK; 2 Department of Plant Sciences, University of Oxford, South Parks Road, Oxford OX1 3RB, UK; 3 Quadram Institute, Rosalind Franklin Road, Norwich Research Park, Norwich NR4 7UQ, UK; 4 Department of Physiology, Anatomy and Genomics, University of Oxford, Sherrington Building, Parks Road, Oxford OX1 3PT, UK; 5 Oxford Ion Channel Initiative, University of Oxford, Oxford OX1 3PT, UK; 6 Department of Metabolism, Digestion and Reproduction, Imperial College, South Kensington Campus, London SW7 2AZ, UK; 7 Department of Experimental Psychology, University of Oxford, Anna Watts Building, Radcliffe Observatory Quarter, Woodstock Road, Oxford OX2 6GG, UK; 8 School of Human Development and Health, Faculty of Medicine, University of Southampton, Southampton SO16 6YD, UK; 9 Department of Pharmacology, University of Oxford, Mansfield Road, Oxford OX1 3QT, UK

**Keywords:** Molecular physiology, Neuroscience, Microbiology

## Abstract

We have shown previously that prebiotic (Bimuno galacto-oligosacharides, B-GOS®) administration to neonatal rats increased hippocampal NMDAR proteins. The present study has investigated the effects of postnatal B-GOS® supplementation on hippocampus-dependent behavior in young, adolescent, and adult rats and applied electrophysiological, metabolomic and metagenomic analyses to explore potential underlying mechanisms. The administration of B-GOS® to suckling, but not post-weaned, rats reduced anxious behavior until adulthood. Neonatal prebiotic intake also reduced the fast decay component of hippocampal NMDAR currents, altered age-specific trajectories of the brain, intestinal, and liver metabolomes, and reduced abundance of fecal *Enterococcus* and *Dorea* bacteria. Our data are the first to show that prebiotic administration to rats during a specific postnatal period has long-term effects on behavior and hippocampal physiology. The study also suggests that early-life prebiotic intake may affect host brain function through the reduction of stress-related gut bacteria rather than increasing the proliferation of beneficial microbes.

## Highlights

• B-GOS® prebiotic supplementation early in life has anxiolytic effects
• Anxiolytic effects last until adulthood
• Postnatal B-GOS® supplements changes kinetics of hippocampal NMDARs and synaptic events
• B-GOS® alters biochemical aging of the brain, digestive system, and the liver

## Graphical abstract

**Figure:**
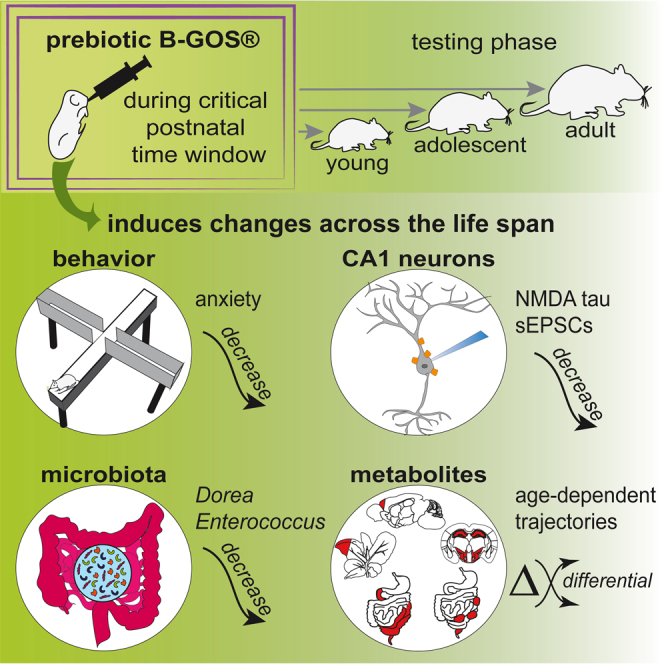

## Introduction

There is increasing evidence of the gut microbiota's influence on the digestive system and brain function of the host. The gut microbiota are established early in life and the changes in their composition in childhood, adolescence and adulthood (Hopkins et al., 2001; Hollister et al., 2015; Agans et al., 2011), suggests an adaptation to age-specific function. The link between gut microbiota and brain function is illustrated in children, whose gut microbiota has been associated with the child's temperament and cognitive performance (Carlson et al., 2018; Christian et al., 2015), and in adults, whose gut microbiota correlate with various neuropsychiatric diseases such as Alzheimer's disease or depression (Grochowska et al., 2018, 2019).

Disrupting the formation of the gut microbiota postnatally, for example, by early exposure to antibiotics can lead to long-term changes in microbiota composition and brain function, measured by anxiety-like behaviors (Leclercq et al., 2017). Germ-free (GF) mice, which lack gut microbiota during development, show impaired emotion-based affect, social cognition, and stress resilience (Sudo et al., 2004, Diaz Heijtz et al., 2011; Desbonnet et al., 2014). If GF mice are conventionalized (*i.e.* when gut bacteria are restored) within the first week after birth, these deficits can be reversed. Conventionalization at later time points, however, is less efficient and virtually ineffective once mice reach adulthood (Cahenzli et al., 2013, Diaz Heijtz et al., 2011; Neufeld et al., 2011a; Clarke et al., 2013). This illustrates that the early establishment of the gut microbiota in the host and their joint postnatal development are key for the gut microbiota to be most beneficial to the host. The plasticity observed during a limited postnatal time period has the potential to shape long-term phenotypes and provides a “therapeutic window” in which deficits can be rectified and health protected long-term.

Growth of beneficial gut bacteria can be promoted through dietary supplements. We and others have demonstrated that the oral administration of prebiotics (substrates that grow beneficial gut bacteria) ameliorated depressive- and anxiety-like behaviors in mice (Burokas et al., 2017; Savignac et al., 2016). We have also shown that, in rats, postnatal administration of a prebiotic (Bimuno^(T)^ galacto-oligosaccahrides, B-GOS®) for three weeks increased protein levels of synaptophysin and the N-methyl-D-aspartate receptor (NMDAR) subunit GluN2A in the hippocampus. These changes were sustained for five weeks after supplementation was ceased when rats were weaned (Williams et al., 2016). GluN2A is important for postnatal brain development with expression levels rapidly increasing within the first three postnatal weeks (Monyer et al., 1994; Sheng et al., 1994; Wenzel et al., 1997). NMDAR signaling in the ventral (anterior) part of the hippocampus has been associated with regulating anxiety (Barkus et al., 2010; Bannerman et al., 2003). We therefore hypothesized that the B-GOS®-induced elevation of hippocampal GluN2A is linked to changes in the anxiety-like behaviors of the rats, which, in turn, were based on gut microbiota induced modifications of hippocampal neuronal NMDAR expression and function. As gut microbiota can affect brain function by inducing changes in the metabolism of the digestive system (Chu et al., 2019; Frohlich et al., 2016; Aoki-Yoshida et al., 2016), the liver (Lv et al., 2019) and the brain (Gacias et al., 2016; Swann et al., 2017; Gao et al., 2018) we also assessed the metabolomes of central and peripheral organs.

Therefore, the aim of this study was to investigate whether the increase of hippocampal network gene expression seen after early-life B-GOS® intake reflected altered brain function. This was achieved by feeding suckling rat pups daily with B-GOS® until weaning, and then (1) examining anxiety-like behavior in weaned, adolescent and adult rats, using the elevated plus maze (EPM); (2) using electrophysiological recordings to measure NMDAR-signaling and synaptic events in ventral hippocampal neurons; (3) exploring gut bacteria composition using the 16S rRNA gene sequencing approach; and (4) assessing the effect of prebiotic and age on the brain and peripheral metabolome using untargeted metabolomic analyzes with proton nuclear magnetic resonance (^1^H NMR) spectroscopy. It is important to investigate the effects of early-life prebiotic supplementation and to confirm whether the effects on brain function can be maintained long-term, thereby possibly imparting resilience to age-related brain disorders.

## Results

### Early-life B-GOS® treatment has anxiolytic effects which persist until adulthood

**Figure 1:**
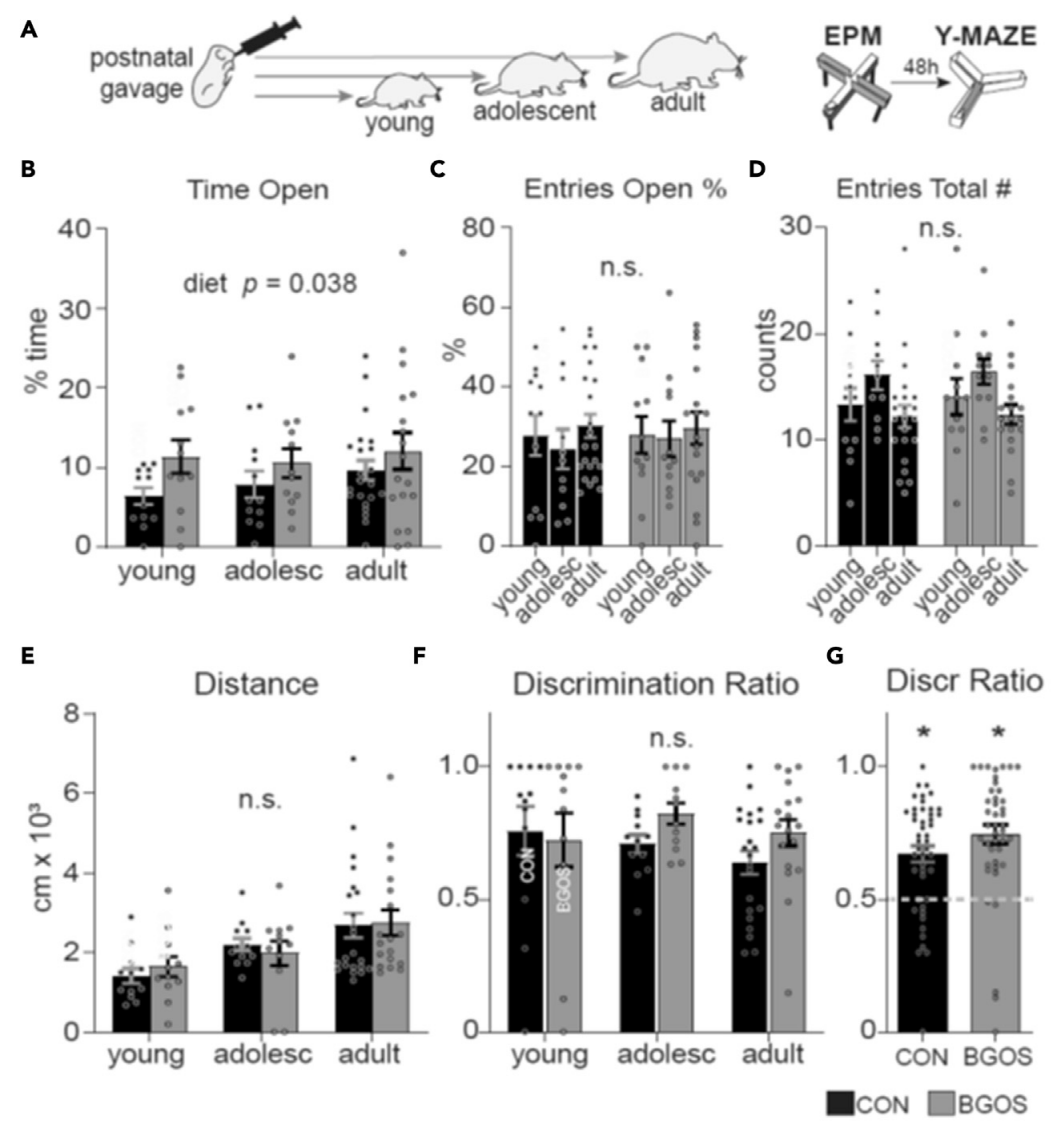
Early-life B-GOS® has long-term effects on anxiety-like behavior but not spatial memory (A) After three weeks of postnatal supplementation with B-GOS® or vehicle, rats were tested when young, adolescent or adult in the EPM to measure anxiety levels and 48 h later in the Y-maze to measure spatial memory. (B) In the EPM, diet had significant effects on time spent in the open arms (p = 0.038). (C) Diet had no effect on entries into the open arms (*F*_*1,81*_ = 0.05, p = 0.827). (D) Diet also had no effect on total entries made into either closed or open arms (*F*_*1,81*_ = 0.15, p = 0.701). (E) nor on distance traveled in the EPM (*F*_*1,81*_ = 0.02, p = 0.902). (F) In the Y-maze, the discrimination ratio was not altered by diet (*F*_*1,82*_ = 1.57, p = 0.214). (G) Same group t-tests for discrimination ratio (Discr Ratio) further confirmed that each group was significantly above ratio expected by chance, indicated dashed line (control and B-GOS®: *p* < 0.0001). *Data are represented as mean +/- SEM. n.s. = not significant, ∗ = significance.*

Male rat pups were fed daily with B-GOS® or control vehicle for the first three weeks of life and then weaned and tested at one of three different developmental stages after weaning: young (3 weeks), adolescent (8 weeks), or adult (4–6 months) (Figure 1A, S1A). Postnatal supplementation had no effect on weight, size or BMI of the animals (Figures S2A–S2D). We first tested the rats in the EPM (Figure 1A). Time spent in open arms was taken as a measure for anxiety levels. Diet had a significant effect on time spent in open arms (*F*_*1, 81*_ = 4.45, p = 0.038), with B-GOS®-fed rats spending more time in the open arms compared to controls. Time spent in open arms was not affected by age (*F*_*2,81*_ = 0.71, p = 0.493) and no interaction effects between diet and age were observed (*F*_*2,81*_ = 0.32, p = 0.727) (Figure 1B). Percentage entries (entries into open arms over total entries) into open arms, however, were not affected by diet (*F*_*1,81*_ = 0.05, p = 0.827), nor were they affected by age (*F*_*2,81*_ = 0.52, p = 0.596) or interactions between diet and age (*F*_*2,81*_ = 0.07, p = 0.929) (Figure 1C).

To rule out locomotion as a factor contributing to the differences in time spent in the open arms, we assessed the number of total entries (in either open or closed arms) and the distance traveled in the maze during the testing phase. Diet had no effect on total entries (*F*_*1,81*_ = 0.15, p = 0.701) or on distance traveled (*F*_*1,81*_ = 0.02, p = 0.902) (Figures 1D and 1E).

To determine whether differences in behavior on the EPM could be explained by differences in spatial exploration in a novel environment, we tested the rats on the Y-maze spatial novelty preference test across the different age groups (Figure 1A, S1A). Neither diet nor age had a significant effect on the discrimination ratio (diet: *F*_*1,82*_ = 1.57, p = 0.214; age: *F*_*2,82*_ = 0.75, p = 0.477; diet × age interaction: *F*_*2,82*_ = 0.86, p = 0.429; Figure 1F). Using single group t-tests on the same data showed that each group performed significantly above what would be expected by chance (*i.e.* a discrimination ratio of 0.5) (control: t_*1,45*_ = 21.49, *p* < 0.0001; BGOS: t_*1,41*_ = 20.83, *p* < 0.0001; Figure 1G), demonstrating a significant memory effect in each group. Furthermore, no differences between groups were observed during the exploratory sample trial phase in the amount of time spent in or entries into either arm (Figures S3A–S3E).

### Anxiolytic effects of B-GOS® are dependent on age at supplementation

**Figure 2:**
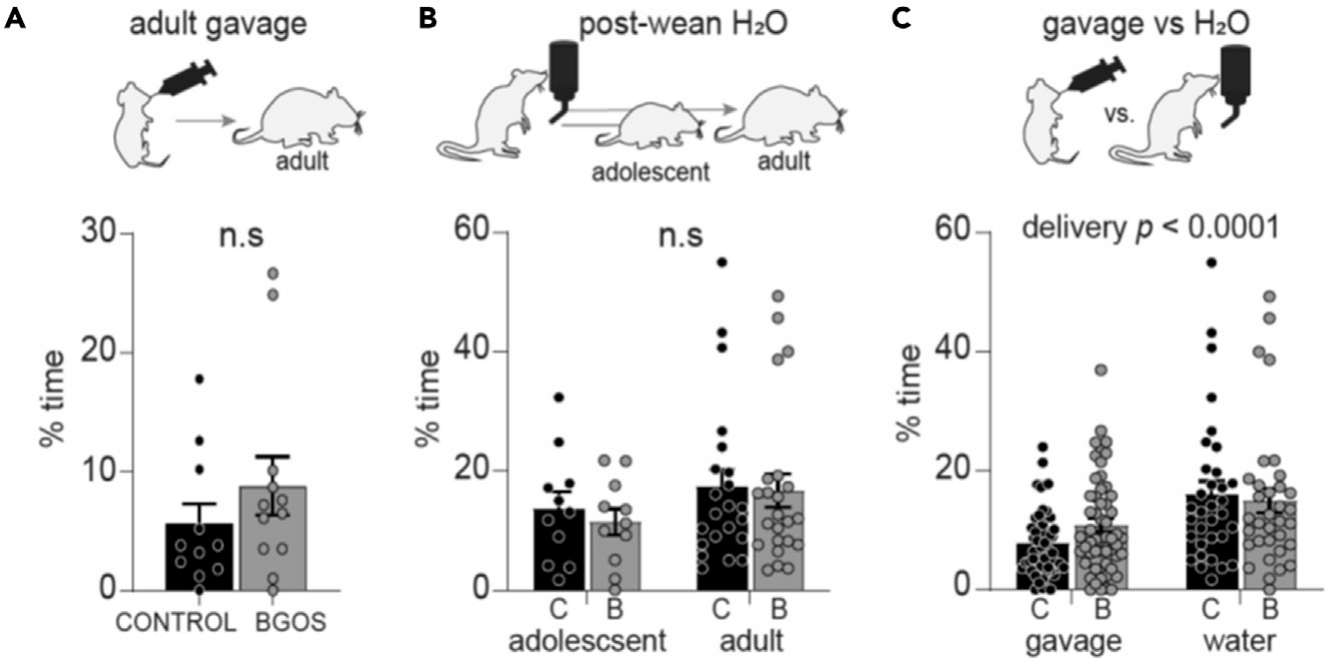
Anxiolytic effects of B-GOS® depend on age at supplementation (A) Rats were gavaged with B-GOS® or vehicle for three weeks during adulthood. Diet groups did not differ in time spent in open arms (p = 0.304). (B) In the post-wean study, another cohort of rats received supplements in the drinking water after weaning for 5.5 weeks. Diet groups did not differ in time spent in the open arms (p = 0.645). (C) Gavage animals were pooled (including postnatal and adult gavage) and compared to rats that received supplements in the drinking water after weaning. The delivery mode (gavage *vs*. water) significantly affected time spent in open arms (*p* < 0.0001). *Data are represented as mean +/− SEM. n.s. = not significant. C = control, B = B-GOS®.*

We next tested whether the anxiolytic effects of B-GOS® depended on age of supplementation. We gavaged adult rats with B-GOS® or vehicle for three weeks and tested them in the EPM at approximately 3 months (Figure 1B). Time spent in open arms did not differ between diet groups (*t*_*21*_ = 1.05, p = 0.304; Figure 2A).

To avoid confounding effects of gavage-induced stress which might have impacted on the adult gavage cohort, we tested another cohort of adult rats in the EPM which received supplements in the drinking water after weaning. Rats were tested either as adolescents at P60, after receiving supplementation continuously from P21 or as adults, 1–2 months after supplementation had ceased (Figure 1C). Diet did not affect time spent in open arms (*F*_*1,63*_ = 0.22, p = 0.645), nor did age (*F*_*1,63*_ = 2.02, p = 0.161, Figure 2B).

To ascertain whether the stress induced by gavage negatively affected the rats' performance in the EPM we compared time spent in open arms of all gavaged rats (postnatal and adult) with the one of the rats that received supplements in the drinking water after weaning. Delivery mode (gavage or water) significantly affected time spent in open arms (*F*_*1,174*_ = 19.30, *p* < 0.0001; Figure 2C).

### B-GOS® alters hippocampal CA1 NMDA receptor kinetics and synaptic events

**Figure 3:**
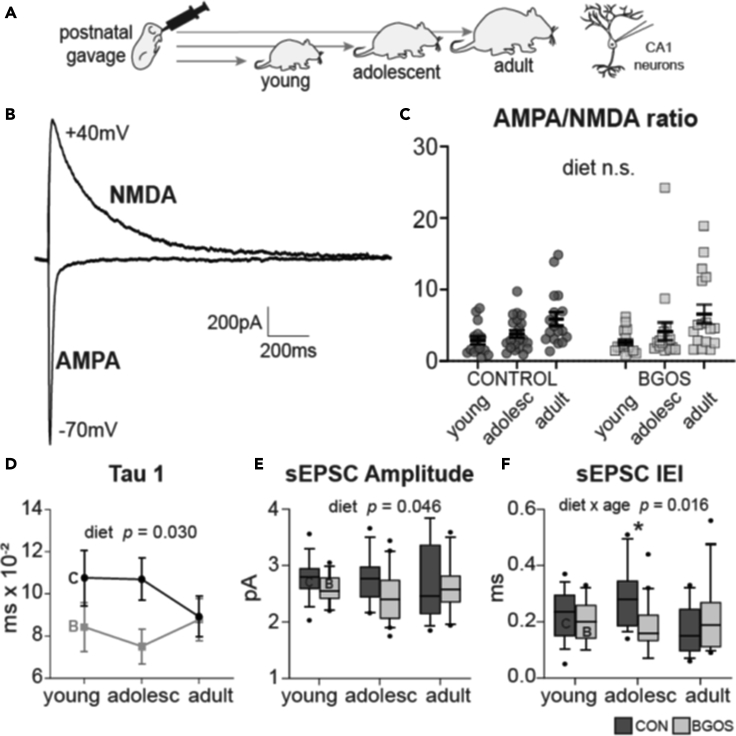
B-GOS® alters kinetics of NMDAR-mediated currents and spontaneous synaptic events of hippocampal CA1 neurons (A) After three weeks of postnatal supplementation with B-GOS® or vehicle, CA1 neurons were whole-cell patch-clamped from young, adolescent or adult rats in the ventral hippocampus. (B) Representative averaged traces of AMPAR- and NMDAR-mediated eESPCs. (C) Diet had no effect on AMPA/NMDA ratio (p = 0.719) but age did (p = 0.001). (D) Double-exponential fittings of the NMDAR-mediated responses revealed that decay time constant (tau) was affected by diet only for the fast component tau-1 (p = 0.030). (E) sEPSC amplitude was significantly affected by diet (p = 0.046). (F) sEPSC IEI was not significantly affected by diet or age but their interaction (p = 0.016), and was more frequent in adolescent B-GOS® rats. *Data are represented as mean +/− SEM. C = control, B = B-GOS®, n.s. = not significant, ∗ = significance after Tukey multiple comparison post-hoc test.*

To test if changes in anxiety-like behavior observed in the B-GOS®-fed rats were linked to altered neuronal signaling, whole-cell patch-clamp recordings were made from cornu ammonis (CA1) pyramidal neurons of the ventral hippocampus from young, adolescent or adult animals supplemented with placebo or B-GOS® (Figure 3A, S1A). Excitatory postsynaptic currents were evoked (eEPSCs) to deduce the postsynaptic AMPA/NMDA receptor ratio (Figure 3B). AMPA/NMDA receptor ratios were not affected by diet (*F*_*1,98*_ = 0.13, p = 0.719), but were affected by age (*F*_*2,98*_ = 7.13, p = 0.001), becoming increasingly larger with age. No interaction effect between diet and age were observed (*F*_*2,98*_ = 0.14, p = 0.868; Figure 3C). We assessed the size of the NMDAR-mediated response by fitting a double exponential to measure amplitudes (A1 and A2) and decay time constants (tau-1 and tau-2). NMDAR amplitude was not affected by diet (A1: *F*_*1,98*_ = 0.87, p = 0.352; A2: *F*_*1,98*_ = 1.39, p = 0.241) or age (A1: *F*_*2,98*_ = 0.27, p = 0.761; A2: *F*_*2,98*_ = 0.47, p = 0.625) and no interaction effects between diet and age were observed (A1: *F*_*2,98*_ = 1.49, p = 0.231; A2: *F*_*2,98*_ = 0.02, p = 0.982; Figures S4D and S4E). Tau-1, however, the fast component of the decay time, was significantly affected by diet (*F*_*1,98*_ = 4.83, p = 0.030) and shortened in B-GOS®-fed rats. Tau-1 was not affected by age (*F*_*2,98*_ = 0.242, p = 0.786) nor by interaction effects (*F*_*2,98*_ = 1.15, p = 0.322; Figure 3D). Tau-2, the slow component of the decay time, was unaffected by diet (*F*_*1,98*_ = 0.27, p = 0.604), age (*F*_*2,98*_ = 2.29, p = 0.107) and interaction effects (*F*_*2,98*_ = 0.46, p = 0.630; Figure S4B). Weighted tau T_W_ also remained unaffected by diet (*F*_*1,98*_ = 0.01, p = 0.923), age (*F*_*1,98*_ = 2.51, p = 0.086) or interaction effect between diet and age (*F*_*1,98*_ = 0.36, p = 0.699; Figure S4C).

We next recorded spontaneous excitatory postsynaptic currents (sEPSCs) in the CA1 pyramidal neurons and measured event amplitudes and their inter-event-intervals (IEI), the latter reflecting event frequency. sEPSC amplitude was significantly affected by diet, being reduced in B-GOS®-fed animals (*F*_*1,91*_ = 4.09, p = 0.046), but remained unaffected by age (*F*_*2, 91*_ = 0.24, p = 0.795) or interaction effects between diet and age (*F*_*2,91*_ = 0.83, p = 0.440; Figure 3E). sEPSC IEI was not affected by diet (*F*_*1,91*_ = 1.91, p = 0.171) or age (*F*_*2,91*_ = 0.96, p = 0.387), but there was a significant interaction effect between diet and age (*F*_*2,91*_ = 4.35, p = 0.016). Tukey multiple comparison post-hoc test revealed that IEIs were significantly smaller (*i.e.* events occurred more frequently) in adolescent B-GOS® animals compared WITH adolescent control rats (p = 0.026; Figure 3F).

### Postnatal B-GOS® supplementation does not alter fecal gut microbiota overall but has effect on two genera

**Figure 4:**
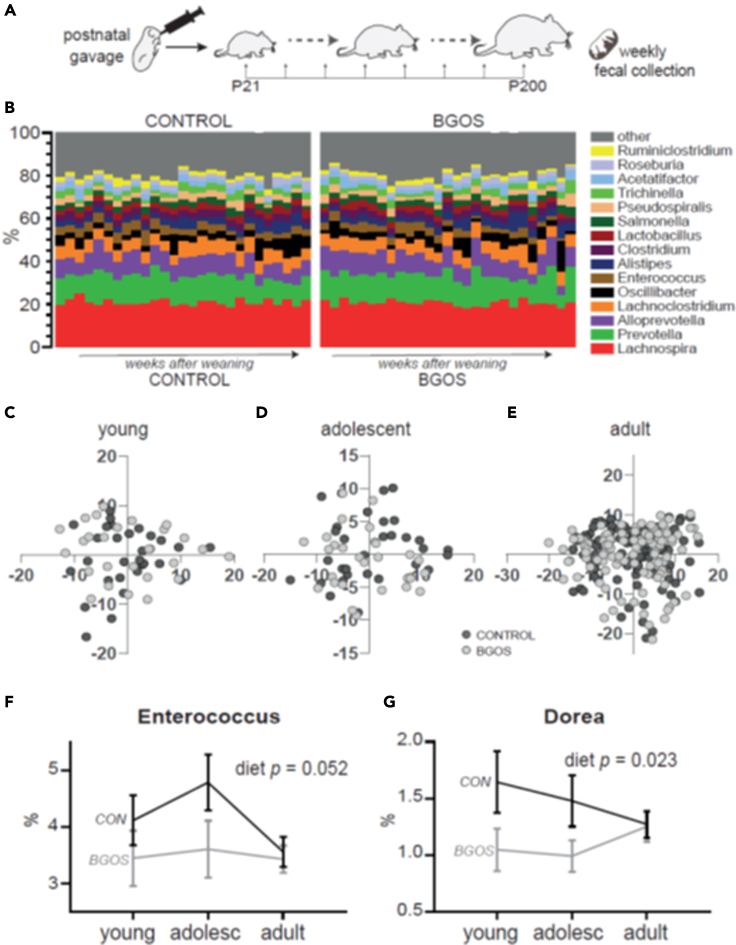
Postnatal B-GOS® supplementation does not affect community structure of fecal gut microbiota on genus level but lowers Enterococcus and Dorea genera (A) After three weeks of postnatal supplementation with B-GOS® or vehicle, feces was collected weekly from day of weaning to six months, for 16S rRNA sequencing. (B) Visual representation of most abundant microbial taxa on the genus level for control and B-GOS® groups. (C–E) PCoA plots of fecal microbiota on genus level of (C) young, (D) adolescent and (E) adult rats. Each data point represents DNA of fecal samples pooled from one rat box. (F) The Enterococcus genus was significantly affected by diet when data was normally distributed after SQRT-transformation (p = 0.048). Graph depicts raw data (p = 0.052). (G) The Dorea genus, too, was significantly affected by diet when data was normally distributed after LOG-transformation (p = 0.013). Graph depicts raw data which still reaches significance (p = 0.003). *Data are represented as mean +/− SEM. CON = control, BGOS = B-GOS®.*

**Figure 5:**
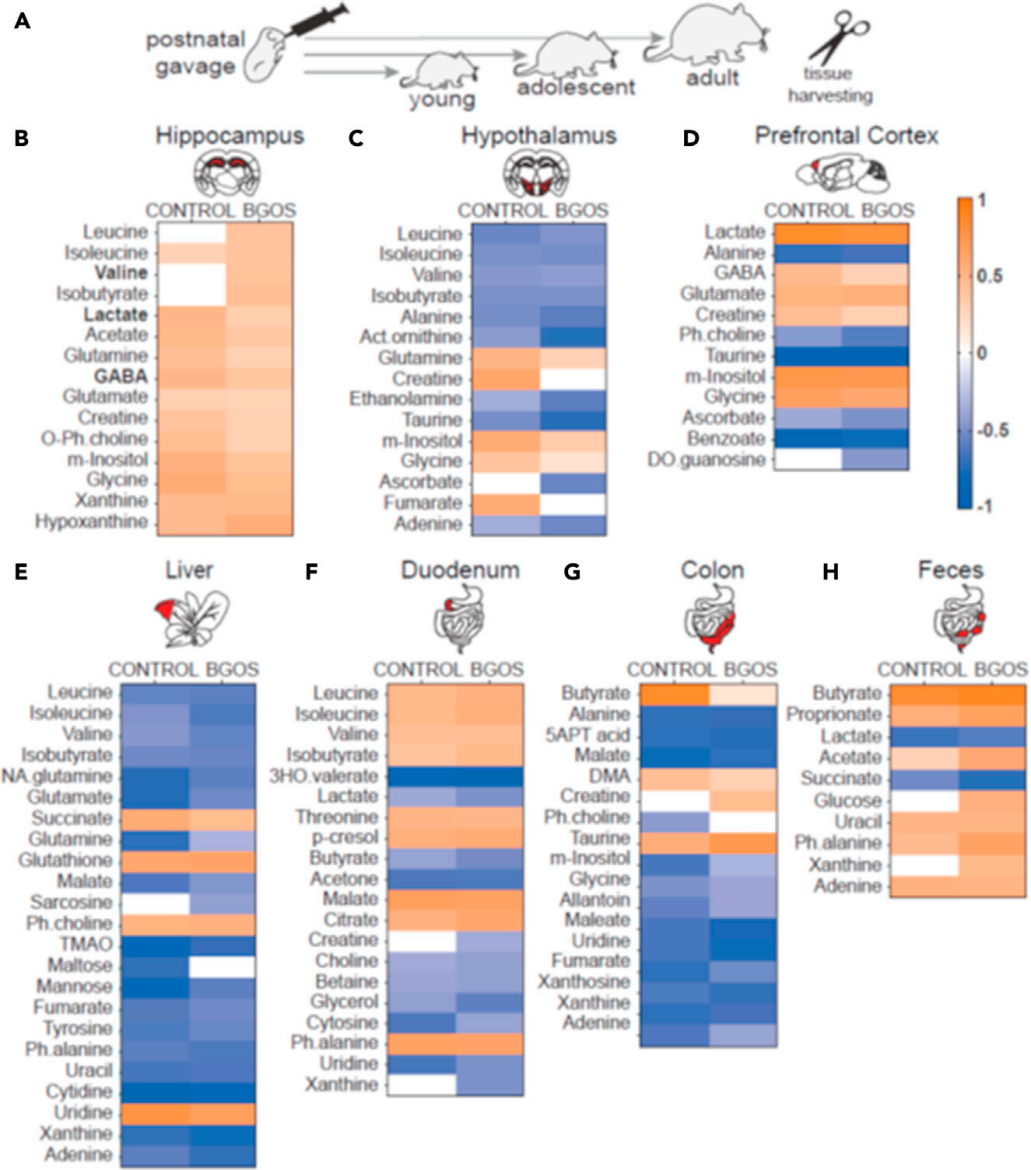
Age-dependent trajectories of metabolomic profiles of brain and peripheral tissues are altered by postnatal B-GOS® supplementation (A) After three weeks of postnatal supplementation with B-GOS® or vehicle, brain and peripheral tissue was harvested from young, adolescent or adult rats. (B–H) Heatmaps represent the correlation coefficients (R) obtained from the significant OPLS models identifying metabolic variation associated with age. Colors indicate the direction of the correlations: metabolites significantly increased with age (orange), significantly decreased with age (blue) or remained unchanged across age groups (white). *Abbreviations: O-Ph.choline - phosphocholine; m-Inositol - myo-Inositol; Act.ornithine - acetyl-ornithine; DO.guoanosine - deoxyguanosine; NA.glutamine - N-acetylglutamine; TMAO - trimethylamine N-oxide; Ph.alanine - phenylalanine; 3HO.valerate - 3-hydroxyisovalerate; 5APT acid - 5-aminopentanoic acid; DMA - dimethylamine*.

To test whether B-GOS®-induced changes in hippocampal physiology and anxiety-like behavior are associated with modifications of the composition of the gut microbiota, we collected feces weekly after weaning until rats reached six months of age for metagenomic analysis (Figure 4A, S1A). We established the composition of the gut microbiota on taxonomic zOTU, order, family and genus level, which we compared between diet groups. In both groups, the two most prevalent bacterial taxa belonged to the Firmicutes and the Bacteroidetes phyla, indicative of a healthy gut. The most prevalent taxa belonging to Firmicutes were Costridiales on order level (Figure S5B), which in turn were dominated by Lachnospiraceae on family level (Figure S5C) and *Ruminococcacea* and *Lachnospira* on genus level (Figure 4B). The most abundant Bacteroidetes were Bacteroidales on order level (Figure S5B), which in turn were dominated by Prevotellaceae on family level (Figure 5C) and *Prevotella* on genus level (Figure 4B).

Using PERMANOVA, we found diet had no effect on the community structure of the taxonomic taxa analyzed (*i.e.* on zOTU, order, family, genus level) (genus: F_*pseudo, 1.320*_ = 0.76, p = 0.763; see Figures 5B and 5C for other p values). Age, however, affected the general community on all taxonomic levels (genus: F_*pseudo, 1.320*_ = 3.2, p = 0.001), as well as the relative abundances. Screening the genus level, we found *Intestinimonas*, *Ruminiclostridium*, *Blautia,* and *Lactobacillus* genera (all part of the Firmicutes phyla) were significantly affected by age (Figures S6B–S6E), as were *Alistipes* (Bacteroidetes phyla) (Figure S6G) and *Acinetobacter* (Proteobacteria phyla) (Figure S6F). When we mapped the fecal microbiota for each age group separately on the genus level using PCoA, we found no differences in clustering between control or B-GOS®-fed animals when rats were young, adolescent or adults (Figures 4C–4E). Screening individual genera, however, revealed that *Enterococcus* and *Dorea*, both part of the Firmicutes phyla, were significantly affected by diet. Both genera were skewed in raw data format and had to be SQRT-transformed (*Enterococcus*) or LOG-transformed (*Dorea*) to restore normality to the data. Diet had significant effects on *Enterococcus* which became evident when transformed data were analyzed (SQRT: *F*_*1, 320*_ = 3.9, p = 0.048, LOG: *F*_*1, 320*_ = 3.9, p = 0.049) while for raw data, the test statistic came close to significance (F_*1, 320*_ = 3.9, p = 0.052; Figure 4F). Diet also had significant effects on the *Dorea* genus which reached significance levels in raw (F_*1, 320*_ = 9.0, p = 0.003), SQRT- (F_*1, 320*_ = 6.1, p = 0.014) and LOG-transformed format (F_*1, 320*_ = 6.3, p = 0.013; Figure 4G).

### Postnatal B-GOS® supplementation shifts age-related changes in the metabolome of central and peripheral tissues

We next examined whether B-GOS® supplementation affected the metabolic profile of the brain and peripheral organs across the age groups (Figure 5A, S1A). First, we assessed whether diet changed the metabolome of the hippocampus which may be a mechanism through which B-GOS® alters behavior. Using age-corrected CA-PLS and PCA analysis, we found that diet had no effect on the metabolome of the hippocampus, nor of other brain areas (hypothalamus, prefrontal cortex). Likewise, diet did not affect the metabolomes of the digestive system (duodenum, colon, colonic feces) or the liver (data not shown, see Figure S7A for Q^2^Ŷ and p values).

However, the metabolome for each tissue was significantly affected by age (see Figures S7A and S7B for Q^2^Ŷ and p values). We therefore created coefficient plots of each tissue and compared the R-values of the tissue-specific metabolites for each diet group across age (see Tables S1A–S1G for R-values and p values). While the majority of metabolites underwent age-related changes consistent in both diet groups, a number of metabolites seemed stable across the age groups in a diet-group specific manner. In the hippocampus, metabolites increased with age with the exception of leucine, valine, and isobutyrate, which remained unchanged in the control group (Figure 5B). In the hypothalamus, B-GOS® rats showed no age-related increase of creatine and fumarate and controls did not show an age-related decrease of ascorbate (Figure 5C). In the prefrontal cortex, controls did not show an age-related decrease of deoxyguanosine (Figure 5D). In the liver, controls did not show an age-related decrease of sarcosine while B-GOS® animals did not show changes in maltose (Figure 5E). Creatine remained stable across age in the duodenum and the colon of control animals (Figures 5F and 5G). Control animals did not show an age-related decline of xanthine in the duodenum and B-GOS® animals did not show an age-related decline of phosphocholine and butyrate in colon (Figures 5F and 5G). In the feces, control animals did not show an age-related increase of glucose and xanthine (Figure 5H) (see Table 1 for overview).

**Table 1:** Metabolites changing with age depending on diet

BGOS +		CONTROL +		BGOS -		CONTROL -	
HPC	leucine valine isobutyrate	HTH	creatine fumarate	HTH	ascorbate	LIV	maltose
COL	Creatine	COL	butyrate	PFC	deoxyguanosine	COL	phosphocholine
FEC	Glucose			LIV	sarcosine		
				DUO	creatine xanthine		

Metabolites listed in BGOS columns change with age only in animals that received B-GOS® postnatally, while metabolites listed in CONTROL columns change with age only in control and not in B-GOS® animals in which these metabolites remained stable. + = age-related increase, — = age-related decrease.

HPC, hippocampus; COL, colon; FEC, intestinal feces; HTH, hypothalamus; PFC, prefrontal cortex; LIV, liver; DUO, duodenum.

## Discussion

This study has shown that supplementing suckling rat pups daily for three weeks with the prebiotic B-GOS® has long-term anxiolytic effects in the EPM. The prebiotic also altered ventral hippocampal CA1 pyramidal signaling and modified age-related metabolic changes of central and peripheral tissues. Although B-GOS® did not have an overall effect on the fecal microbiota it did change two genera of the Firmicutes phyla.

The anxiolytic action of postnatal B-GOS® supplementation was not accompanied by a change in locomotion in the EPM. Furthermore, there was no effect on spatial exploration in a novel environment or on short-term memory when tested in the Y-maze spatial novelty preference test. These observations suggest that the prebiotic-mediated changes of behavior in the EPM were due to lowered levels of anxiety and not differences in locomotion, exploratory behavior or spatial memory. The observation that this effect was only observed when prebiotic supplementation occurred before weaning is consistent with our previous studies and those of others. That is, prebiotic supplementation in adult mice had no effect on anxiety-like behavior in the light-dark box (Savignac et al., 2016) or the EPM (Burokas et al., 2017). The latter study found reduced anxiety-like behaviors in the open field test, which was not included in the current investigation. This suggests that the anxiolytic effects of prebiotics are most potent when administered during a critical postnatal period.

Postnatal B-GOS® supplementation also caused alterations in neuronal signaling in the ventral hippocampus, an area known to regulate anxiety (Bannerman et al., 2003; Barkus et al., 2010). The age-related increase of AMPA/NMDA receptor ratios in CA1 neurons is consistent with age-dependent decrease in NMDAR-signaling while AMPAR signaling remains relatively stable (Wenk and Barnes, 2000; Crescenzi et al., 2017). The prebiotic altered the kinetics of the NMDAR-evoked responses by shortening tau-1, the fast component of the decay time, without changing time constant T_W_. Changes in NMDAR subunit composition (reviewed by Cull-Candy and Leszkiewicz, 2004) and/or differences in phosphorylation rates may explain these findings. It has been shown that changes in the ratio of protein kinase A (PKA) to calcineurin can affect NMDAR currents (Raman et al., 1996). When dephosphorylated by calcineurin, residues Ser-900 and Ser-929 on the C-terminus of the GluN2A subunit can reduce the decay time of NMDAR currents (Townsend et al., 2004; Maki et al., 2013). Further pharmacological experiments are required to confirm whether B-GOS® affects these channel properties.

While GluN2A receptors have mostly been associated with neuronal NMDARs located in the synapse, GluN2A receptors have also been detected in astrocytes *in vitro* (Lee et al., 2010; Zhou et al., 2010; Szychowski and Gminski, 2019; Conti et al., 1996). It is therefore conceivable that the elevated protein levels of hippocampal GluN2A we observed previously (Williams et al., 2016) were driven by non-neuronal cells. This would explain why overall NMDAR-evoked changes were not affected by B-GOS® supplementation in CA1 pyramidal neurons. The B-GOS®-induced changes in glial cells may also be upstream of the observed altered neuronal network activity. Spontaneous synaptic events of CA1 pyramidal neurons were smaller in B-GOS®-fed animals. Increased synaptic event amplitude in CA1 neurons has been previously seen in mice undergoing a fear conditioning learning paradigm (Zhou et al., 2009), suggesting that a reduced amplitude may reflect less anxiety-like behavior. Whether B-GOS® induces changes in non-neuronal cells which consequently affect synaptic signaling of CA1 neurons requires investigation.

The intake of B-GOS® did not alter the overall composition of the gut microbiota when assessed on zOTU, order, family and genus level. In previous studies we have demonstrated that the administration of this prebiotic to young adult rats increased fecal Bifidobacteria and reduced the abundance of other genera that were not affected in the present investigation (Savignac et al., 2013; Kao et al., 2018). However, the fecal microbiota from animals administered with B-GOS® at adolescence and adulthood in the present study were not analyzed and so it is difficult to conclude whether there are age-dependent differential effects of prebiotic intake on gut microbial communities. Nevertheless, we have now shown that two genera, *Enterococcus* and *Dorea*, were affected by diet. An increase of *Enterococcus*, a conditional pathogen, in response to chronic unpredictable mild stress has been reported in adult mice (Sun et al., 2019). It is known that gavage is a source of stress in rodents which can increase anxiety levels (Balcombe et al., 2004; Jones et al., 2016). Similarly, our rats showed increased levels of anxiety-like behavior in the EPM when compared with the cohort that received supplements in the drinking water. It is therefore conceivable that the lower levels of *Enterococcus* in the B-GOS®-fed mice is a sign of increased resilience to the stress posed by the postnatal gavage which resulted in lowered anxiety-like behavior in the EPM. Sun and colleagues (Sun et al., 2019) also reported a negative correlation between *Enterococcus* levels and time spent in open arms of the EPM. The *Dorea* genus, however, has not, to our knowledge, been reported to correlate with anxiety levels. An increase of the *Dorea* genus is found in diabetes (Li et al., 2020) and in rodents fed a high-fat diet (Guo et al., 2020; Velazquez et al., 2019). Various strains of the *Dorea* genus have been implicated as markers of insulin resistance and inflammation (Brahe et al., 2015). The lower levels of *Dorea* in our B-GOS®-fed rats may indicate a more robust gut health which might be tied in with increased resilience to the postnatal stress. However, further studies are necessary to elucidate the role of the *Dorea* genus in anxiety regulation.

We also observed age-specific changes in various genera with an age-related increase in *Intestinimonas* and *Alistipes*, an age-related decrease of *Acinetobacter* and a noticeable difference in adolescent animals in the abundances of *Ruminiclostridium*, *Blautia*, *Lactobacillus* genera. It remains to be determined whether a spike in the abundance of these three genera may be typical of adolescence also in humans.

It is noteworthy that GF mice, which develop without any gut microbiota, can also display lower levels of anxiety-like behavior (Diaz Heijtz et al., 2011; Luo et al., 2018; Clarke et al., 2013). These animals are often hyperactive (Diaz Heijtz et al., 2011; Luo et al., 2018) which might distort measures of anxiety. Anxiety in rodents is important for self-preservation and limits exposure to potentially dangerous stimuli. It is possible that a lack of gut microbiota during development impairs the adoption of these self-preserving strategies. Comparing the average time spent in open arms of our B-GOS®-fed rats (35 s/11.8%) to reports of GF mice (70 s - Diaz Heijtz et al., 2011, 38% - Neufeld et al., 2011b) reveals a significantly lower level of anxiety in GF, and so may be considered as a behavioral impairment. The paradox that both the absence of gut bacteria, and prebiotic nurturing of beneficial bacteria are both anxiolytic, may be the result of removing or reducing, respectively, the influence of pathogenic bacteria that might underlie anxious behaviors.

Prebiotic intake influenced age-specific shifts in the metabolomes of the brain, the digestive system and the liver. In the brain, B-GOS® supplementation prevented an age-dependent increase of creatine in the hypothalamus. Creatine metabolites have been shown to affect GABA receptors (de Deyn and Macdonald, 1990; Neu et al., 2002) and supplementation with creatine can reduce depressive-like behaviors in mice (Cunha et al., 2012; Pazini et al., 2016). The hypothalamus is part of the hypothalamus-pituitary-adrenal axis and thus involved in regulating stress and anxiety-like behaviors. In the hippocampus, an age-related increase in leucine, valine and isobutyrate was seen in B-GOS®-fed rats. Leucine-containing dipeptides have been shown to have anti-depressant-like effects and can suppress stress activation of the hypothalamo-pituitary-adrenal axis (Mizushige et al., 2020). Valine, leucine, and isoleucine are branched chain amino acids which activate the mTOR pathway (Xu et al., 2001; Zhou et al., 2018). Hyperactivity of the mTOR pathway (through PTEN knockout) has been shown to reduce anxiety-like behavior in the EPM (Lugo et al., 2014). The age-related increase in leucine and valine in B-GOS®-fed rats may activate the mTOR pathway which may contribute to maintaining low levels of anxiety long-term. In the digestive system, B-GOS® supplementation resulted in an age-dependent decrease in xanthine in the duodenum and an increase in the fecal excretion of xanthine possibly reflecting an age-related decrease in xanthine uptake in the gut. Xanthine inhibits adenosine receptors and their administration has been shown to increase anxiety-like behavior in the EPM (Imaizumi et al., 1994; Kulkarni et al., 2007). Notably, the short-chain fatty acid butyrate remained stable in B-GOS® treated animals while it increased in controls. Colonic butyrate levels have been shown to increase in response to stress (Maltz et al., 2019). The lack of increase in B-GOS® rats might be indicative of their lower stress levels in response to their postnatal experiences.

Whether these shifts in age-dependent metabolic regulation contributes to maintaining lower anxiety levels in B-GOS®-fed rats warrants further investigation.

In summary, supplementation of B-GOS® had anxiolytic effects on rat behavior but only when given during the critical postnatal period. The anxiolytic effects of early-life B-GOS® supplementation can be sustained long-term through prevailing cellular changes in the ventral hippocampus, presumably evoked by the gut microbiota, although the fecal microbial profiles were not affected on order level. Prebiotic supplementation altered the biochemical aging of the brain and periphery which may also contribute to the enduring anxiolytic effects of the prebiotic into adulthood. These results have important implication of early-life interventions with prebiotics to ensure long-term brain health and emotion regulation. This is especially relevant as complex prebiotics are found in human breast milk (human milk oligosaccharides, HMOs). HMOs are lacking from infant formula, which is used by more than half of mothers in Britain from 6 weeks after birth (Public Health England, 2019). Infant formula may provide a vehicle through which prebiotics can be delivered at an early age to ensure healthy brain development and emotion regulation which is important for generalized anxiety disorder as well schizophrenia, obsessive-compulsive disorder and depression (reviewed by Hofmann et al., 2012).

### Limitations of the study

The main limitations of this study are that the observed anxiolytic effects of B-GOS® are specific to the EPM. Further assays using various paradigms to measure anxiety levels in rodents will be required to determine whether the anxiolytic effects B-GOS® exerts are universal to all testing conditions. Another notable drawback of the study is that it was limited to male rats, in line with previous studies (Williams et al., 2016), and female rats may have displayed different responses within the parameters measured. Therefore, the findings of this study cannot be generalized to both sexes, and other studies with female animals, or parallel exploration of both sexes, are required. Unfortunately, given the number of experimental measures conducted practical and cost issues limited the current investigation to one sex.

## STAR★Methods

### Key resources table

**Table:** 

REAGENT or RESOURCE	SOURCE	IDENTIFIER
**Chemicals, peptides, and recombinant proteins**		

Bimuno-galactooligosaccharide (B-GOS®)	Clasado BioSciences Ltd, UK	EAN: 5060143340000
Kynuretic acid	Sigma-Aldrich	Cat# K3375g
Gabazine	Tocris	Cat# 1262
Cesium methanesulfonate	Sigma-Aldrich	Cat#C1426
QX-314	Tocris	Cat# 1014

**Critical commercial assays**		

Qiaquick PCR Purification Kit	Qiagen	Cat# 28104

**Oligonucleotides**		

Primers: V4 fragment of 16s rRNA gene: GTGCCAGCMGCCGCGGTAA	(Caporaso et al., 2012)	515F
Primers: V4 fragment of 16s rRNA gene: GGACTACHVGGGTWTCTAAT	(Caporaso et al., 2012)	806R

**Deposited data**		

RNA-seq data	This paper	accession: PRJEB39202; https://www.ebi.ac.uk/ena/browser/home

**Software and algorithms**		

EthoVision XT 11.5	Noldus	https://www.noldus.com/ethovision-xt
Primer6	PRIMER-e	https://www.primer-e.com/our-software/
IMPaCTS	(Ritler et al., 2019) DOI: 10.1038/s41598-019-56073-y	https://github.com/csmsoftware/IMPaCTS
Matlab R2018a	Mathworks	https://uk.mathworks.com/products/matlab.html
SPSS Statistics 25	IBM	https://www.ibm.com/uk-en/products/spss-statistics
Prism 8	Graphpad	https://www.graphpad.com/scientific-software/prism/
Usearch10	Drive5	https://www.drive5.com/usearch/

### Resource availability

#### Lead contact

Further information and requests for resources should be directed to and will be fulfilled by the lead contact, Dr Phil Burnet (phil.burnet@psych.ox.ac.uk).

#### Material availability

This study did not generate any unique reagents.

### Method details

#### Animals

All experimental procedures were performed in accordance with the UK Home Office Animals (Scientific Procedures) Act (1986) and Home Office guidelines and approved by the Local Animals Welfare and Ethical Review Body (AWERB) at the University of Oxford. Sprague-Dawley (SD) rats were purchased from Harlan Laboratory, UK, as litters (consisting of lactating dam with 4-6 male pups aged P2 or as male adults. Animals were kept under standard controlled laboratories conditions (12 h light–dark cycle, lights on at 7 a.m., 21 ± 1 °C, humidity 50 ± 5%). All animals received standard rat chow *ad libitum*.

#### Supplementation

*Postnatal supplementation* (Experiments 1, 3, 4 and 5) (Figure 1A): littermates were randomly assigned to control or prebiotic diet (B-GOS) groups aiming at equal numbers per group. All pups received supplements through gavage using bent steel feeding needles, daily from P3 to P21 with either the Bimuno® supplement (Clasado BioSciences Ltd, UK) or a control sugar solution. The supplement consisted of 50% Bimuno-galacto-oligosaccharides (B-GOS®), 27% lactose, 13% glucose and 11% galactose. A final dose of 0.5g/kg/day B-GOS® prebiotic (1g/kg/day Bimuno® supplement) was chosen for consistency with our previous work (Kao et al., 2018). The control solution contained lactose, glucose and galactose (Sigma-Aldrich, UK) in water at the same proportions as in the Bimuno® supplement. Gavage supplementation ensured consistent levels of prebiotics in the B-GOS® diet group independent of maternal care and hierarchy within the litter. As prebiotics are naturally found in the dam's mother's milk, pups of the control groups, which were gavaged with the sugar solution, also received some amount of prebiotic, however, to a significantly lesser degree. Rats were weaned at P21 and housed in separate B-GOS® and control groups to avoid microbial cross-colonization. On P22 animals were either used for testing, culled for tissue or kept for later time points in groups of 2–5 animals (Figure S1A).

*Adult gavage supplementation* (Experiment 2) (Figure 1B): rats were purchased as young, male adults (250 g) and, after at least 1 week of acclimatization, were gavaged daily for 3 weeks (B-GOS®, 0.5g/kg/day) followed by testing in the EPM (Figure S1B).

*Post-wean water supplementation* (Experiment 2) (Figure 1C): another cohort of rats was purchased as suckling dams with male pups. Pups were left to suckle without supplementation until weaning at P21, when rats were randomly assigned to control or B-GOS® diet groups and housed in groups of three. B-GOS® was given continuously in the drinking water at a concentration that delivered a final dose of 0.5 g/kg/day until P60. Water intake was monitored by weighing water bottles every two days, when bottles were replaced and B-GOS® concentration adjusted to maintain dosage. Rats in control groups received standard drinking water without supplements, as control sugars in the drinking water have been shown to have adverse effects on behavioral testing due to increased water intake (Gronier et al., 2018). Rats were tested either on P60, 0 days post supplementation (dps) or between one and three months after supplementation had ceased (30–90 dps) and rats were between P90 and P120 of age (Figure S1C).

#### Behavioral tests

Anxiety levels were measured in the elevated plus maze task (EPM) (Gastambide et al., 2015) and spontaneous spatial novelty preference was assessed in the Y-maze to evaluate rapidly acquired short-term spatial memory (Bannerman et al., 2008). Wooden EPM (74.5 cm above ground) consisted of two opposing open arms and two opposing closed arms (61 × 10 cm each) separated by a central area (10 × 10 cm). At the beginning of each trial, rats were placed into the central area of the maze, facing an open arm, and allowed to explore the maze for 5 min. Time spent in open or closed arms, entries into open or closed arms and distance traveled were measured.

Wooden Y-maze was made of clear plastic, has three identical arms (each 49 × 17 × 27 cm) which were pseudo-randomly assigned to ‘start’, ‘familiar’ and ‘novel’ arm for each rat. In the initial 5 min sample trial phase, rats were allowed to explore start and familiar arms whilst the novel arm remained closed off by an opaque Perspex screen. After completion of the sample trial phase, rats were placed into the home cage for 1 min and then put back into the maze for the 2 min ‘test phase’ during which all three arms were accessible. Time spent in familiar or novel arm was measured and a discrimination ratio (time in novel arm/(time in novel + familiar arms)) was calculated.

EthoVision XT 11.5 was used to video-track the animals in the maze and to measure time spent in arms (EPM: open, closed; Y-maze: novel, familiar) as well as distance traveled (EPM). Each animal was tested at one specific time point only (young, adolescent, adult) to avoid training effects. Groups consisted of 12 animals each in young controls and B-GOS® groups, 12 animals each in adolescent control and B-GOS® groups and 22 control adult and 18 B-GOS® adult animals (to account for the longer time span and the possible impact of variation in age.) [NB: Owing to technical issues the number of entries into closed or open arms in the EPM was lost for one control adolescent rat]. Postnatally gavaged rats were tested first on the EPM and 48 hours later on the Y-Maze. Adult gavaged rats as well as rats which received supplements in the drinking water were only tested on the EPM.

#### Electrophysiology

For electrophysiological recordings, new cohorts of rats were used which received postnatal supplementation and did not undergo behavioral testing to avoid confounding effects. Rats were anaesthetized with isoflurane before being culled though dislocation of the neck when young (P22-P25), adolescent (P56-P59) or adult (P128-P199). Brains were removed and placed in warmed sucrose buffer containing (in mM): NaCl 95, NaHCO_3_ 26, KH_2_PO_4_ 1.2, KCl 1.8, CaCl 0.5, MgSO_4_ 7, glucose 15, sucrose 50 and kynuretic acid (1 mM), with pH 7.3–7.4 when saturated with carbogen gas (95% O_2_ and 5% CO_2_). Coronal slices containing the ventral hippocampal formation (350 μm) were prepared using a vibratome (Leica Vibratome VT1200S), the CA3 and CA1 dissected, and the slices maintained in a submerged chamber at 37°C, saturated with carbogen gas (95% O_2_ and 5% CO_2_). After recovery for at least one hour, slices were transferred to submerged recording chamber, and superfused at 5–8 ml min^−1^ with aCSF at 32–34°C, perfused with carbogen gas, containing 10 μM gabazine to block GABAergic synaptic transmission. *Visualized whole-cell voltage-clamp recordings* were obtained from CA1 pyramidal cells using patch pipettes filled with an internal solution containing (in mM) cesium methansulfonate 140, NaCl 5, HEPES 10, EGTA 0.2, MgATP 2, NaGTP 0.3, QX-314 5 (all chemicals were obtained from Tocris Bioscience and Sigma-Aldrich) and 1% biocytin (wt/vol), with series resistance and whole-cell capacitance compensated by ∼55% using Multiclamp 700B software (Molecular Devices). All slices were fixed overnight in 4% PFA and biocytin-filled cells labeled with streptavidin-conjugated fluorescent markers. All patched cells were examined and pyramidal-cell specific morphology confirmed.

*Spontaneous EPSCs were* recorded at −70 mV and synaptic events detected using template matching (Clements and Bekkers, 1997). Amplitude and inter-event intervals (IEI) were analyzed by calculating the running median. *Evoked EPSCs* were quantified by placing a monopolar tungsten stimulation electrode in the stratum radiatum and responses recorded at −70 mV (AMPA) and +40 mV (NMDA; after the decay of AMPAR-mediated currents at 50 ms). Evoked responses were averaged over 6–10 sweeps and peaks used to determine AMPA/NMDA ratio. A double exponential was fitted to NMDA-evoked responses to measure NMDA amplitude and time constant T by calculating weighted time constant T_W_ (Cathala et al., 2000). Amplitude-1 and tau-1 represent the fast decay component, while amplitude-2 and tau-2 represent the slow decay component. Data were low-pass filtered at 3 kHz and acquired at 10 kHz. Recordings were amplified and digitized using an Axon Multiclamp700B amplifier and an ITC-18 A/D board (Instrutech) respectively. Data acquisition and stimulation protocols were carried out using custom-written procedures in IgorPro (WaveMetrics) and experiments in which series resistance changed by more than 25% were excluded.

#### Metagenomics

For metagenomics experiment, feces was collected weekly from animals which had received postnatal supplementation, from 0 to 25 weeks after weaning (when postnatal supplementation was ceased at P21). Depending on rat age and pellet size, 2–10 pellets were collected from each rat box containing at least two rats and immediately frozen and maintained at −80°C until DNA extraction. Each week, between 3 and 9 such samples were collected per diet group from different cohorts to minimize cage and cohort effects. Metataxonomic profiling was performed on samples for each week from week 0 (P21-24, then each new week counted after 7 days from P25) to week 25 (P193-200) with 4–8 samples from different cohorts per week.

To extract DNA, samples were combined with guanidine hydrochloride (4mM) and mechanically homogenized using a tissue lyser (TissueLyser LT, Qiagen) followed by centrifugation. Supernatant was combined with silica dioxide solution (0.1 g/ml) and again centrifuged. Pellets were subsequently washed in EtOH/NaCl (70% EtOH including 100mM NaCl) and then dried. DNA was collected as supernatant after the pellet was resuspended in nano-filtered water, briefly heat activated (55°C for 1 min) and again centrifuged.

The V4 region of the 16S rRNA gene was amplified and sequenced (Illumina MiSeq) from the extracted DNA to obtain a global representation of the microbiome composition. V4 region primers: 515F: GTGCCAGCMGCCGCGGTAA and 806R: GGACTACHVGGGTWTCTAAT using two-sided barcode “Golay's” system (Caporaso et al., 2012). PCR conditions were as follows: 20 μl reaction volume with 4 μl Phusion HF buffer (New England Biolabs), 0.4 μl dNTPs, 1 μl forward and 1 μl reverse primers (10 pmol each), 1 μl of template DNA (5 ng/μl) and 0.2 μl Phusion polymerase using 30 cycles at 98°C (15 sec), 55°C (15 sec) and 72°C (30 sec) with the final extension of 7 min at 72°C. PCR products were purified using Qiaquick PCR Purification Kit (Qiagen) and sent for sequencing using Illumina Miseq using reagent kit v3 (300 pair-end).

Reads were merged using *usearch v10 fastq_mergepairs* and quality filtered using *fastq_maxee* with EE set to 1.0. All reads longer or shorter than the expected 292bp+/−2bp were discarded. Samples were standardized to 2500 reads each in order to obtain 329 samples passing this threshold (control: 30, 32, 130, B-GOS®: 30, 32, 130) for each age group. Reads were binned into ASV-level zero-radius OTUs (zOTUs) with chimera removal using *Usearch10 unoise3* command (Edgar, 2016). zOTUs were annotated using SILVA v132 database. Genus, family and order level annotation is based on the zOTU alignment results. Data were used to visualize principal coordinate analysis (PCoA) plots as well as to run two-way PERMANOVA type III with 9999 permutations using a two-factor design (diet and age (‘young’, ‘adolescent’, ‘adult’) and diet X age). Data were analyzed using PRIMER6 software (PRIMER-e) based on the Bray-Curtis similarity matrix of sample-to-sample community similarity. See supplementary methods for details.

#### Metabolomics

For metabolomics experiment, a new cohort of rats was supplemented postnatally and not used for behavioral testing. From young (3 weeks), adolescent (8 weeks) or adult (4.5–6 months) animals the following tissues were collected: brain (prefrontal cortex, hypothalamus, hippocampus), large intestine (distal end), duodenum, feces (extracted from colon) and liver. 5–8 animals were used for tissue harvesting for each diet group at each time point. All tissue was frozen immediately at −80°C until extraction of polar metabolites. For extraction, tissue samples were homogenized in a chloroform-methanol mix (2:1) using a tissue lyser (Precellysis 24, Bertin Technologies). Homogenates were combined with 300 μl water, vortexed and spun (13,000 *g* for 10 min) to separate the aqueous and organic phases. Water and methanol were removed from the aqueous phase by using a vacuum concentrator (SpeedVac). Samples were stored at −80°C (brains and organs) or analyzed immediately (feces). For analysis, extracted tissue samples were reconstituted in phosphate buffer (pH 7.4) in 100% D_2_O containing 1 mM of the internal standard, 3-(trimethylsilyl)-[2,2,3,3,-^2^H_4_]-propionic acid (TSP). Fecal pellets were homogenized in 700 μl phosphate buffer, centrifuged at 13,000 *g* for 15 min and supernatant transferred to 5mm NMR tubes. The metabolic profiles were measured using ^1^H nuclear magnetic resonance (NMR) spectroscopy and analyzed as described previously (Beckonert et al., 2007): One dimensional 600 MHz ^1^H NMR spectra were acquired on a Bruker NMR spectrometer (Bruker Biospin GmbH, Rheinstetten, Germany) using a standard one-dimensional solvent suppression pulse sequence (relaxation delay, 90° pulse, 4 μs delay, 90° pulse, mixing time, 90° pulse, acquire FID). For each sample, 32 transients were collected in 64K frequency domain points with a spectral window set to 20 ppm. A relaxation delay of 4 s, a mixing time of 10 ms, an acquisition time of 2.73 s and 0.3 Hz line broadening was used. Spectra were referenced to the TSP resonance at δ 0.0. Spectral phasing and baseline correction were automatically performed using Topspin 3.2 (Bruker Biospin GmbH, Rheinstetten, Germany). Raw NMR spectra were digitized, aligned and normalized using the Imperial Metabolic Profiling and Chemometrics Toolbox (IMPaCTS) (https://github.com/csmsoftware/IMPaCTS) in MATLAB (Version, 2018a; Mathworks Inc). After digitization of the spectra, redundant peaks (TSP, H_2_O and urea) were removed, followed by manual alignment of the spectra to reference peaks using a recursive segment-wise peak alignment approach (Veselkov et al., 2009). Aligned spectra were normalized by probabilistic quotient normalization (Dieterle et al., 2006).

*For metabolomics analysis*, multivariate data analysis was performed based on mathematical modeling using the IMPaCTS toolbox in Matlab R2018a (MathWorks) (https://github.com/csmsoftware/IMPaCTS). For principal component analysis (PCA) pareto scaling applied to first visualize the global variance of the data sets to reveal intrinsic similarities in the spectral profiles and to identify outliers (only one outlier was detected and removed in the hippocampal data set). Orthogonal projection to latent structures (OPLS) analysis was performed on mean-centered data. OPLS models were constructed to identify differences in spectra between diets and age groups. ^1^H NMR spectroscopic profiles were used as the descriptor matrix (X) and age groups (young, adolescent, adult) or diet (control, B-GOS®) were used as the predicted variable (Y). Orthogonal signal correction filters were used to remove variation of the descriptor matrix unrelated to prediction variable. Loading coefficient plots were generated by back-scaling transformation to display the covariance between the Y-prediction matrix and the signal intensity of the metabolites in the NMR data (X). Signal direction and magnitude relate to the covariation of the metabolites with the Y-response in the model. Plots were color-coded to identify metabolites with significant correlation coefficients (R^2^) between each metabolite and the Y-prediction variable. R^2^_max_ was used to construct heatmaps. The predictive performance (Q^2^Ŷ) of the model was calculated using a seven-fold cross validation approach, model validity was established by permutation testing (1000 permutations) and the results are given as p-values with a value of 0.05 or less as the cutoff for significance. To adjust for confounding factors such as age, covariate-adjusted-projection to latent structures (CA-PLS) models were also constructed using ^1^H NMR metabolic profiles (Posma et al., 2018). Model robustness was assessed using Monte-Carlo cross-validation using a total of 100 models, for the 24,706 centered and scaled spectral variables. A *p*-value was calculated for each variable on the basis of 25 bootstrap re-samplings of the data in each of the 100 models, to estimate the variance and the mean coefficient across the 100 models. Spectral variable importance was assessed with the false discovery rate q-value, with a value of ≤ 0.05. See supplementary methods for details.

### Quantification and statistical analysis

Behavioral and electrophysiology data were analyzed with SPSS Statistics 25 (IBM) and Prism 8 (GraphPad). Diet groups (*B-GOS® vs control*) were compared using Student’s t-test. All results were confirmed when nonparametric tests were applied. Comparisons of groups (*B-GOS® vs control*) across time points (*young vs. adolescent vs. adult*) were performed using univariate ANOVA with two dependent variables (*diet + age*). If data were skewed, square-root transformation and/or logarithm transformation was applied which restored normality. ANOVA results did not differ when applied to raw and square-root transformed data, with the exception of three genera analyzed for metagenomics (Figure 4F and S6D, S6E). In these three cases, significance was not reached when data were analyzed in raw format and data were skewed. SQRT and LOG-transformation restored normality and both led results reaching significance. All graphs depict raw data unless stated otherwise (Figures S6D and S6E). Bar and line graphs with error bars represent mean values with standard error of the mean. Box plots showing electrophysiology results for Amplitude and IEI of spontaneous EPSCs (Figures 3E and 3F) plot hinges at 10^th^ and 90^th^ percentile and median as midline. Whiskers represent minimum and maximum values. Significance level alpha was defined as p value < 0.05, represented with an asterisk in plots.

## Data Availability

• RNA-seq data have been deposited at the EMBL-EBI ENA short-read archive database and are publicly available as of date of publication. Access number and link are listed in the key resources table. • Source codes for NMR spectra analysis have been deposited to the public repository GitHub as well as Zenodo. The links and DOI are provided in the key resource table. • All data and code can be requested from the lead contact.
