# Supplementary material for: Postnatal prebiotic supplementation in rats affects adult anxious behaviour, hippocampus, electrophysiology, metabolomics, and gut microbiota
Source: iScience. 2021 Sep 10;24(10):103113. doi: 10.1016/j.isci.2021.103113 (PMC8476651; doi:10.1016/j.isci.2021.103113)
Supplement: Document S1. Figures S1–S7 and Table S1 [file mmc1.pdf]

iScience, Volume *24*

Supplemental information

Postnatal prebiotic supplementation in rats affects adult anxious behaviour, hippocampus, electrophysiology, metabolomics, and gut microbiota

Sonia O. Spitzer, Andrzej Tkacz, Helene M. Savignac, Matthew Cooper, Natasa Giallourou, Edward O. Mann, David M. Bannerman, Jonathan R. Swann, Daniel C. Anthony, Philip S. Poole, and Philip W.J. Burnet

Supplementary Information

Animals & Supplementation

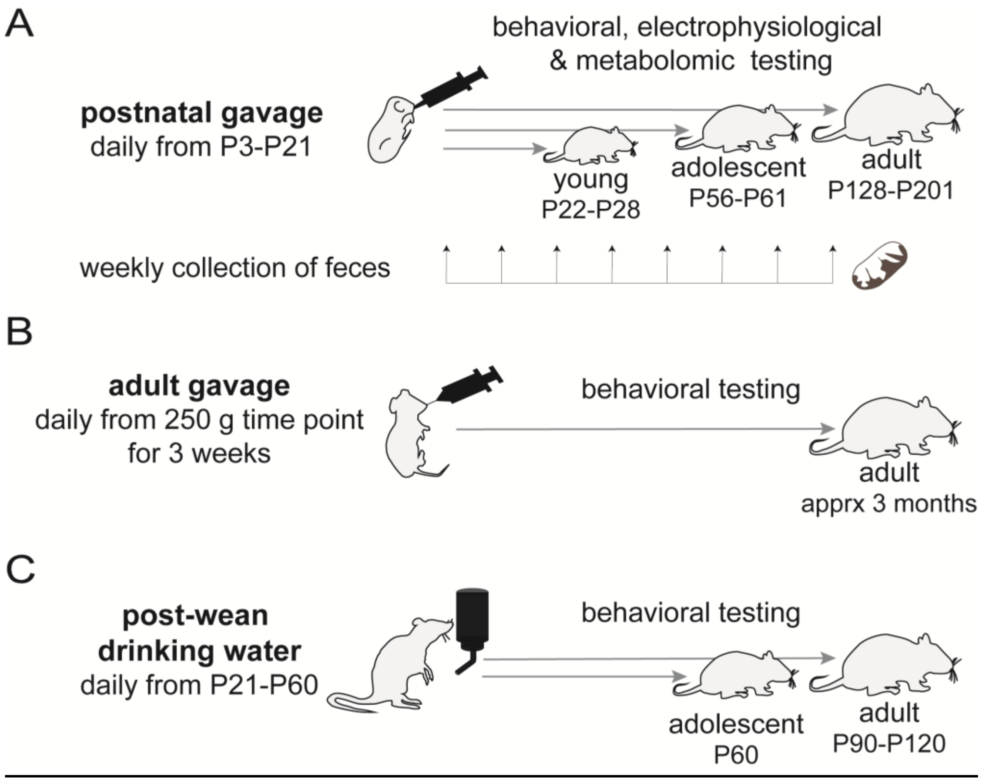

Figure S1. **Overview of supplementation strategies. Related to STAR Methods.**

A) *Postnatal gavage*: for experiment 1, 3, 4 and 5, male rat pups were gavaged for three weeks before behavioral, electrophysiological and metabolomics experiments were conducted when rats were young, adolescent or adult. Feces for RNA sequencing was collected weekly after weaning at P21 until rats were 6 months old. **B**) *Adult gavage:* for experiment 2, rats were purchased when adults (250g) and gavaged for three weeks before behavioral experiments were conducted. **C**) *Post-wean drinking water:* for experiment 2, rats received supplementation in the drinking water after weaning from P21 to P60. Rats were tested either on P60, 0 days post supplementation (dps) or between one and three months after supplementation had ceased (30 – 90 dps) when rats were between P90 and P120 of age.

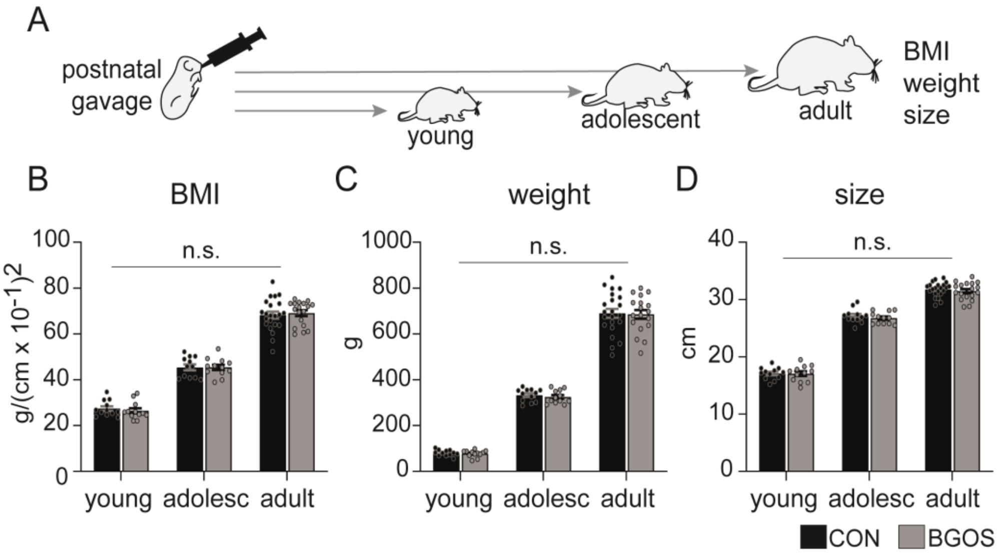

Figure S2. Weight and size of rats was not affected by postnatal supplementation with B-GOS®. Related to Figure 1.

A) After three weeks of postnatal supplementation, rats were measured and weighed when young, adolescent or adults. B-D) Diet had no effect on B) BMI ($F_{1,82}$ = 0.0003, p = 0.986), C) weight ($F_{1,82}$ = 0.14, p = 0.709) or D) size ($F_{1,82}$ = 0.62, p = 0.433), and no interaction effects between age and diet were observed (BMI: $F_{2,82}$ = 0.22, p = 0.801; weight: $F_{2,82}$ = 0.01, p = 0.989; size: $F_{2,82}$ = 0.12, p = 0.884). N-numbers: young: 12 control & B-GOS®, adolescent: 12 young & B-GOS®, adult: 22 control, 18 B-GOS®. *Data are represented as mean +/- SEM. n.s. = not significant. BMI = body-mass-index.*

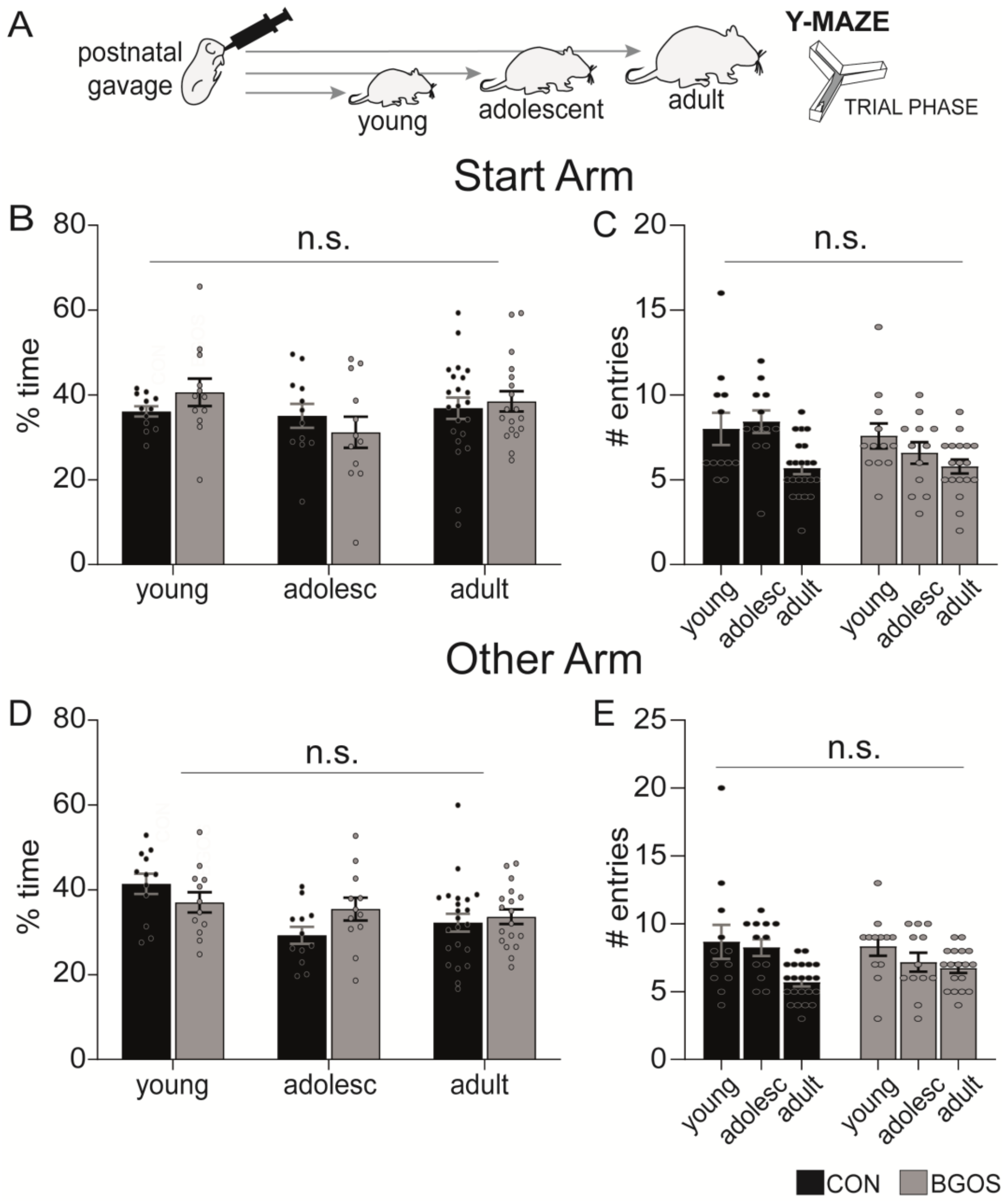

Figure S3. Early-life B-GOS® has no effects on performance in the Y-maze during the sample trial phase. Related to Figure 1.

A) After three weeks of postnatal supplementation with B-GOS® or vehicle, rats were tested at young, adolescent or adult time points the Y-maze spatial novelty test, starting with a 5 min sample trial phase during which randomly assigned 'start' and 'other' arm were accessible. B-C) Diet had no effect on B) time spent in start arm ($F_{1,82}$ = 0.10, p = 0.749), or C) entries into start arm ($F_{1,82}$ = 2.12, p = 0.149). D-E) Diet had no effect on D) time spent in other arm ($F_{1,82}$ = 0.33, p = 0.568) or E) entries into the other arm ($F_{2,82}$ = 0.06, p = 0.811). *Data are represented as mean +/- SEM. n.s. = not significant. n-numbers: young: 12 control & B-GOS®, adolescent: 12 control & B-GOS®, adult: 22 control, 18 B-GOS®*

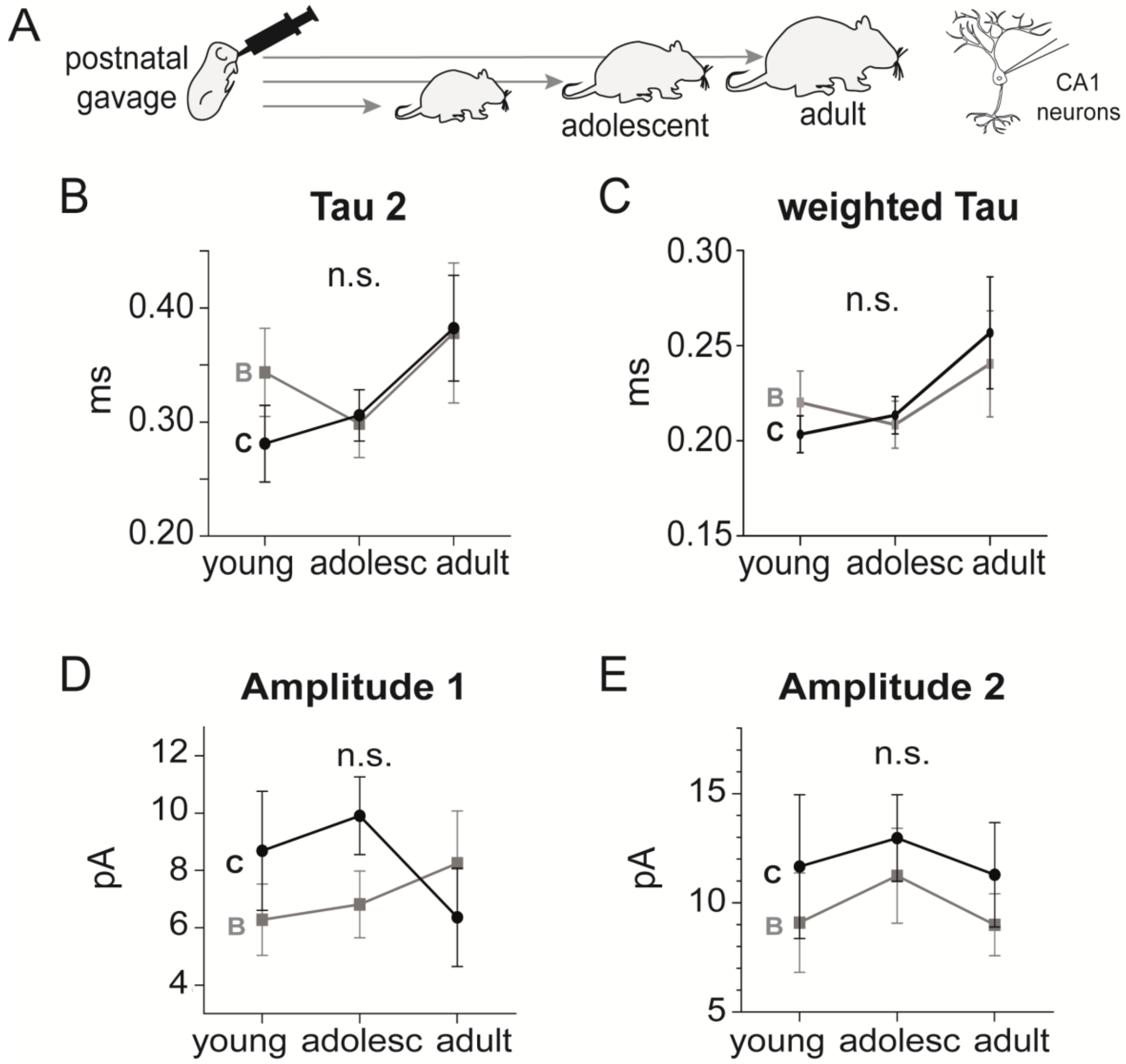

Figure S4. Early-life B-GOS® does not affect slow tau component or amplitude of NMDA currents of CA1 neurons. Related to Figure 3.

A) After three weeks of postnatal supplementation with B-GOS® or vehicle, CA1 neurons were whole-cell patch-clamped from young, adolescent or adult rats in the ventral hippocampus. B+C) Double-exponential fittings of the NMDAR-mediated responses revealed that the slow component tau-2 of the decay time constant (B) was not affected by diet (p = 0.604), nor was weighted tau (p = 0.923; C). D+E) Postnatal diet did also not affect amplitude-1 (p = 0.352; D), or amplitude-2 (p = 0.241; E) of NMDA currents. *Data are represented as mean +/- SEM. n.s. = not significant. C = control, B = B-GOS®. n-numbers: young: control – 15, B-GOS® – 16; adolescent: control – 21, B-GOS® – 18; adult: control – 17, B-GOS® – 17. n.s. = not significant.*

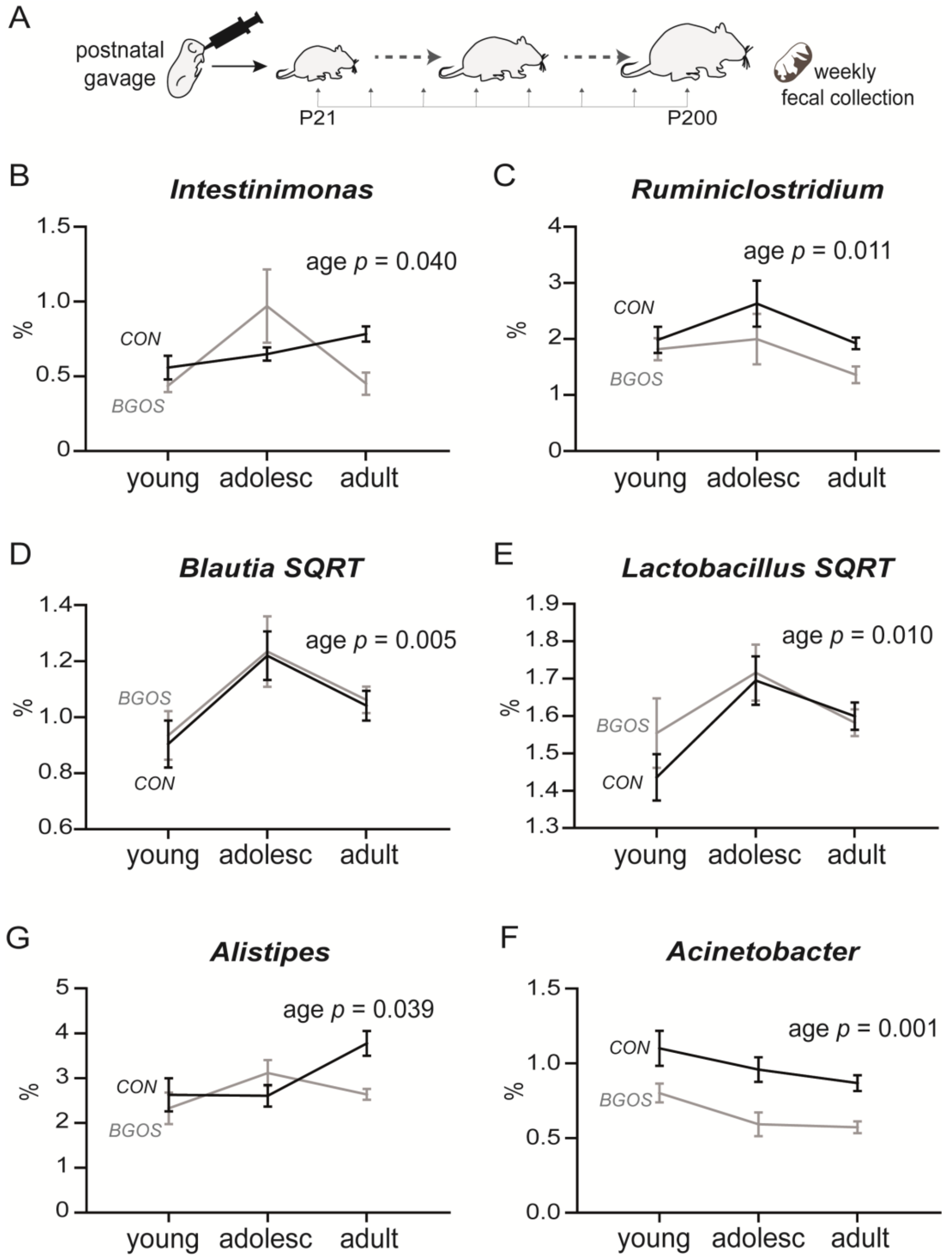

Figure S5. Early-life B-GOS® does not affect gut microbiota on order or family level. Related to Figure 4.

A) After three weeks of postnatal supplementation with B-GOS® or vehicle, feces was collected weekly from day of weaning to six months, for DNA extractions and 16S rRNA sequencing. B-C) Visual representation of the most abundant microbial taxa on the B) order and C) family level, representing ~80% of the community, for each week from weaning to 6 months, separated by diet group. Diet did not affect overall composition of the gut microbiota on order ($F_{pseudo\ 1,320}$ = 0.5, p = 0.904), family ($F_{pseudo\ 1,320}$ = 0.7, p = 0.777) or zOTU level ($F_{pseudo\ 1,320}$ = 1.0, p = 0.510) and no interaction effects were observed. D-E) PCoA plots of fecal samples on D) order and E) family level. Each data point represents DNA of fecal samples of one rat box. No differences in clustering were observed between diet or age groups.

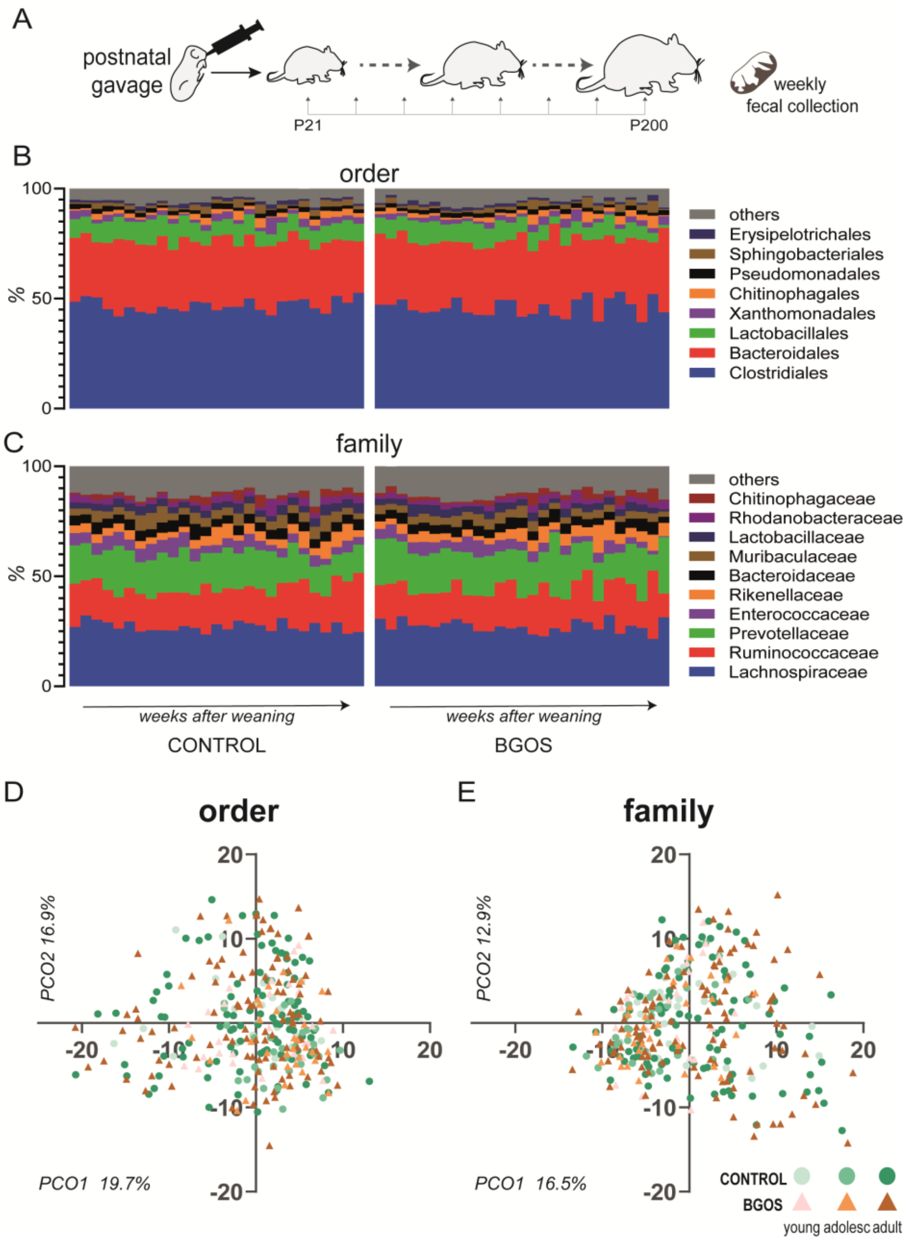

Figure S6. Age, not diet, affected community structure and altered several genera of the Firmicutes, Bacteroidetes and Proteobacteria phyla. Related to Figure 4.

A) After three weeks of postnatal supplementation with B-GOS® or vehicle, feces was collected weekly from day of weaning to six months, for DNA extractions and 16S rRNA sequencing. B-E) Age affected community levels of taxa belonging to the Firmicutes phyla, such as B) Intestinimonas ($F_{2,320}$ = 3.3, p = 0.040), C) Ruminiclostridium ($F_{2,\ 320}$ = 4.6, p = 0.011), D) Blautia ($F_{2,320}$ = 3.3, p = 0.040) and E) Lactobacillus ($F_{2,320}$ = 3.3, p = 0.040). G) Alistipes, part of the Bacteroidetes phyla, was also affected by age ($F_{2,320}$ = 3.3, p = 0.039), as was F) Acinetobacter (Proteobacteria phyla) ($F_{2,320}$ = 5.4, p = 0.005). PERMANOVA revealed an effect of age on the community structure on order ($F_{pseudo,\ 1.320}$ = 3.8, p = 0.0001), family ($F_{pseudo,\ 1.320}$ = 3.6, p = 0.0001) and zOTU level ($F_{pseudo,\ 1.320}$ = 14.6, p = 0.0001). Note that all genera had to be SQRT or LOG-transformed to ensure normality. Significance did not change in raw and transformed data (graphs depict raw data), with the exception of D+E (for which p-values did not reach significance when raw data was analysed with ANOVA). However, both SQRT- and LOG-transformation resolved normality issues and led to age having significant effects (D+E depicted in SQRT-transformed format).

A **Diet or age** used as predictive variable for OPLS analysis.

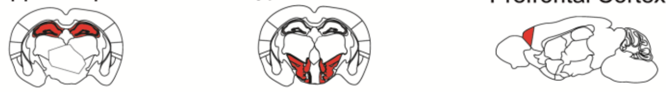

OPLS	Hippocampus *diet*	Hippocampus *age*	Hypothalamus *diet*	Hypothalamus *age*	Prefrontal Cortex *diet*	Prefrontal Cortex *age*
p-value	0.043+	0.001	0.697	0.001	0.487	0.001
$Q^2\hat{Y}$	0.033	0.612	-0.013	0.678	-0.063	0.832

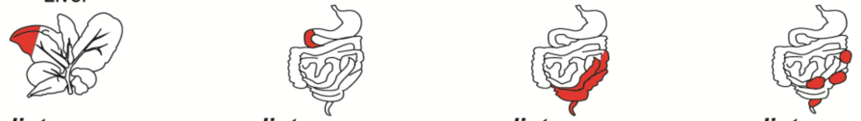

OPLS	Liver *diet*	Liver *age*	Duodenum *diet*	Duodenum *age*	Colon *diet*	Colon *age*	Faeces *diet*	Faeces *age*
p-value	0.009+	0.001	0.153	0.001	0.656	0.001	0.854	0.001
$Q^2\hat{Y}$	0.051	0.738	-0.019	0.575	-0.126	0.730	-0.084	0.372

+*significance was lost when adjusted for age*

B **Age** used as predictive variable for OPLS analysis. Control and B-GOS® groups analysed separately.

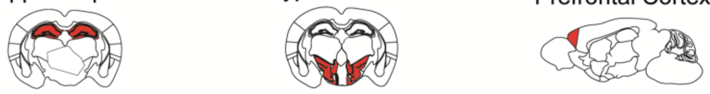

OPLS	Hippocampus *CONTROL*	Hippocampus *BGOS*	Hypothalamus *CONTROL*	Hypothalamus *BGOS*	Prefrontal Cortex *CONTROL*	Prefrontal Cortex *BGOS*
p-value	0.001	0.001	0.001	0.001	0.001	0.001
$Q^2\hat{Y}$	0.599	0.489	0.385	0.714	0.808	0.810

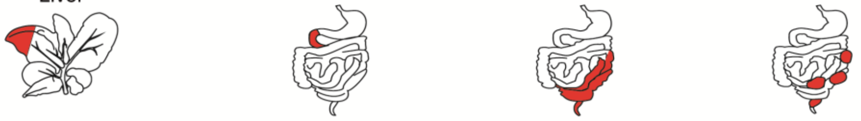

OPLS	Liver *CONTROL*	Liver *BGOS*	Duodenum *CONTROL*	Duodenum *BGOS*	Colon *CONTROL*	Colon *BGOS*	Faeces *CONTROL*	Faeces *BGOS*
p-value	0.001	0.001	0.001	0.001	0.001	0.001	0.001	0.001
$Q^2\hat{Y}$	0.7249	0.6609	0.5641	0.4178	0.7754	0.7957	0.4642	0.6469

Figure S7. Age but not diet affected the overall metabolomic profile of of brain and peripheral tissues. Related to Figure 5

P-values and $Q^2\hat{Y}$ values of OPLS of brain and peripheral tissues with A) diet or B) age as the predictive variable Y. + *significance was lost when adjusted for age*

A

HIPPOCAMPUS

	CONTROL		B-GOS	
	R_{max}	p-value	R_{max}	p-value
Leucine	0.246	0.096	0.394	5.59E-03
Isoleucine	0.289	4.85E-02	0.393	5.73E-03
Valine	0.164	0.271	0.408	3.99E-03
Isobutyrate	0.274	0.062	0.428	2.42E-03
Lactate	0.498	3.68E-04	0.332	2.13E-02
Acetate	0.488	4.97E-04	0.366	1.04E-02
Glutamine	0.436	2.19E-03	0.311	3.13E-02
GABA	0.498	3.63E-04	0.382	7.41E-03
Glutamate	0.324	2.62E-02	0.287	4.79E-02
Creatine	0.405	4.72E-03	0.318	2.75E-02
O-Ph.choline	0.450	1.49E-03	0.312	3.06E-02
myo-Inositol	0.543	7.95E-05	0.409	3.90E-03
Glycine	0.578	2.10E-05	0.461	9.66E-04
Xanthine	0.469	8.87E-04	0.489	4.20E-04
Hypoxanthine	0.466	9.72E-04	0.572	2.20E-05

B

HYPOTHALAMUS

	CONTROL		B-GOS	
	R_{max}	p-value	R_{max}	p-value
Lactate	0.845	8.68E-14	0.799	1.73E-11
Alanine	-0.697	5.37E-08	-0.656	5.62E-07
GABA	0.464	1.01E-03	0.305	3.73E-02
glutamate	0.528	1.38E-04	0.558	4.58E-05
Creatine	0.452	1.42E-03	0.312	3.29E-02
Ph.choline	-0.447	1.63E-03	-0.589	1.34E-05
Taurine	-0.882	2.66E-16	-0.877	6.14E-16
myo-Inositol	0.763	4.37E-10	0.739	2.98E-09
Glycine	0.689	8.37E-08	0.610	5.41E-06
Ascorbate	-0.367	1.12E-02	-0.485	5.50E-04
Benzoate	-0.842	1.18E-13	-0.775	1.55E-10
DOguanosine	-0.266	0.071	-0.453	1.40E-03

C

PREFRONTAL CORTEX

	CONTROL		B-GOS	
	R_{max}	p-value	R_{max}	p-value
Leucine	-0.542	8.45E-05	-0.471	6.34E-04
Isoleucine	-0.492	4.37E-04	-0.494	3.06E-04
Valine	-0.455	1.33E-03	-0.435	1.80E-03
Isobutyrate	-0.493	4.34E-04	-0.485	4.12E-04
Alanine	-0.504	3.00E-04	-0.587	9.29E-06
Act.ornithine	-0.441	1.93E-03	-0.711	1.05E-08
Glutamine	0.517	1.98E-04	0.293	4.07E-02
Creatine	0.622	3.07E-06	0.155	0.286
Ethanolamine	-0.346	1.72E-02	-0.579	1.29E-05
Taurine	-0.509	2.55E-04	-0.725	3.74E-09
m-Inositol	0.590	1.28E-05	0.339	1.74E-02
Glycine	0.388	7.02E-03	0.179	0.218
Ascorbate	-0.226	0.126	-0.542	5.75E-05
Fumarate	0.590	1.29E-05	0.272	0.058
Adenine	-0.353	1.50E-02	-0.530	8.87E-05

D

LIVER

	CONTROL		B-GOS	
	R_{max}	p-value	R_{max}	p-value
Leucine	-0.558	5.61E-05	-0.566	3.35E-05
Isoleucine	-0.473	9.10E-04	-0.613	4.64E-06
Valine	-0.451	1.65E-03	-0.552	5.85E-05
Isobutyrate	-0.532	1.43E-04	-0.545	7.45E-05
N-Acetylglutamine	-0.736	5.68E-09	-0.584	1.66E-05
Glutamate	-0.743	3.39E-09	-0.518	1.91E-04
Succinate	0.579	2.49E-05	0.456	1.28E-03
Glutamine	-0.706	4.20E-08	-0.318	2.94E-02
Glutathione	0.614	5.63E-06	0.638	1.44E-06
Malate	-0.617	4.88E-06	-0.465	9.96E-04
Sarcosine	-0.240	0.107	-0.423	3.02E-03
Phosphocholine	0.522	1.98E-04	0.545	7.48E-05
Trimethylamine N-oxide	-0.806	1.43E-11	-0.714	1.71E-08
Maltose	-0.699	6.44E-08	-0.192	0.195
Mannose	-0.784	1.17E-10	-0.581	1.83E-05
Fumarate	-0.600	1.05E-05	-0.521	1.76E-04
Tyrosine	-0.607	7.72E-06	-0.537	9.87E-05
Phenylalanine	-0.586	1.88E-05	-0.614	4.50E-06
Uracil	-0.634	2.29E-06	-0.616	4.06E-06
Cytidine	-0.839	3.23E-13	-0.791	3.60E-11
Uridine	0.767	4.90E-10	0.693	6.61E-08
Xanthine	-0.691	1.09E-07	-0.762	4.74E-10
Adenine	-0.584	2.08E-05	-0.695	5.89E-08

E

DUODENUM

	CONTROL		B-GOS	
	R_{max}	p-value	R_{max}	p-value
Leucine	0.480	5.61E-04	0.552	5.87E-05
Isoleucine	0.492	3.86E-04	0.547	6.81E-05
Valine	0.432	2.15E-03	0.444	1.79E-03
Isobutyrate	0.388	6.40E-03	0.475	7.35E-04
3HOvalerate	-0.810	3.22E-12	-0.817	2.63E-12
Lactate	-0.366	1.05E-02	-0.482	6.04E-04
Threonine	0.513	1.93E-04	0.496	3.89E-04
p-cresol	0.545	6.12E-05	0.592	1.18E-05
Butyrate	-0.404	4.42E-03	-0.512	2.36E-04
Acetone	-0.623	2.22E-06	-0.615	4.26E-06
Malate	0.690	5.94E-08	0.663	3.78E-07
Citrate	0.538	7.98E-05	0.613	4.55E-06
Creatine	-0.224	0.126	-0.359	1.32E-02
Choline	-0.360	1.20E-02	-0.423	3.08E-03
Betaine	-0.387	6.55E-03	-0.416	3.59E-03
Glycerol	-0.427	2.49E-03	-0.571	2.73E-05
Cytosine	-0.624	2.13E-06	-0.401	5.19E-03
Ph.lalanine	0.652	5.29E-07	0.641	1.20E-06
Uridine	-0.637	1.13E-06	-0.488	5.00E-04
Xanthine	-0.193	0.188	-0.479	6.54E-04

F

COLON

	CONTROL		B-GOS	
	R_{max}	p-value	R_{max}	p-value
Butyrate	0.848	5.34E-14	0.162	0.278
Alanine	-0.697	5.28E-08	-0.716	1.55E-08
5APT acid	-0.705	3.11E-08	-0.720	1.16E-08
Malate	-0.770	2.46E-10	-0.707	2.87E-08
DMA	0.442	1.85E-03	0.308	3.51E-02
Creatine	-0.243	0.100	0.461	1.11E-03
Ph.choline	-0.439	2.00E-03	-0.180	0.226
Taurine	0.568	3.09E-05	0.736	3.70E-09
m-Inositol	-0.630	2.04E-06	-0.312	3.28E-02
Glycine	-0.475	7.43E-04	-0.387	7.16E-03
Allantoin	-0.564	3.64E-05	-0.392	6.43E-03
Maleate	-0.629	2.14E-06	-0.788	4.95E-11
Uridine	-0.635	1.60E-06	-0.770	2.58E-10
Fumarate	-0.705	3.11E-08	-0.514	2.19E-04
Xanthosine	-0.608	5.93E-06	-0.695	5.86E-08
Xanthine	-0.708	2.64E-08	-0.653	6.65E-07
Adenine	-0.617	3.90E-06	-0.397	5.71E-03

G

FECES

	CONTROL		B-GOS	
	R_{max}	p-value	R_{max}	p-value
Butyrate	0.831	2.79E-13	0.883	1.04E-16
Propionate	0.564	3.00E-05	0.673	1.65E-07
Lactate	-0.651	5.58E-07	-0.591	9.74E-06
Acetate	0.292	4.38E-02	0.595	8.11E-06
Succinate	-0.520	1.49E-04	-0.712	1.41E-08
Glucose	0.167	0.258	0.552	4.78E-05
Uracil	0.510	2.14E-04	0.510	2.12E-04
Phenylalanine	0.472	7.03E-04	0.654	4.59E-07
Xanthine	0.272	0.061	0.480	5.54E-04
Adenine	0.531	1.04E-04	0.518	1.62E-04

Tables S1. Quantification of age-related changes of metabolites of brain and peripheral tissues as illustrated in heatmaps in Figure 5 – related to Figure 5.

R-values and p-values of metabolites for control and B-GOS® groups for A-C) brain and D-G) peripheral tissues. Cells highlighted in dark grey show metabolites whose p-values are **not** significant.
